# *SLFL* Genes Participate in the Ubiquitination and Degradation Reaction of S-RNase in Self-compatible Peach

**DOI:** 10.3389/fpls.2018.00227

**Published:** 2018-02-22

**Authors:** Qiuju Chen, Dong Meng, Zhaoyu Gu, Wei Li, Hui Yuan, Xuwei Duan, Qing Yang, Yang Li, Tianzhong Li

**Affiliations:** Laboratory of Fruit Cell and Molecular Breeding, College of Horticulture, China Agricultural University, Beijing, China

**Keywords:** peach, self-compatibility, *S*-locus F-box like gene (*SLFL*), S-RNase, ubiquitination, SCF complex

## Abstract

It has been proved that the gametophytic self-incompatibility (GSI), mainly exists in Rosaceae and Solanaceae, is controlled by *S* genes, which are two tightly linked genes located at highly polymorphic *S*-locus: the *S-RNase* for pistil specificity and the F-box gene (*SFB/SLF*) for pollen specificity, respectively. However, the roles of those genes in SI of peach are still a subject of extensive debate. In our study, we selected 37 representative varieties according to the evolution route of peach and identified their *S* genotypes. We cloned pollen determinant genes mutated *PperSFB1m, PperSFB2m, PperSFB4m*, and normal *PperSFB2*, and style determinant genes *PperS1-RNase, PperS2-RNase, PperS2m-RNase*, and *PperS4-RNase*. The mutated *PperSFBs* encode truncated SFB proteins due to a fragment insertion. The truncated PperSFBs and normal PperSFB2 interacted with PperS-RNases demonstrated by Y2H. Normal *PperSFB2* was divided into four parts: box, box-V1, V1-V2, and HVa-HVb. The box domain of PperSFB2 did not interact with PperS-RNases, both of the box-V1 and V1-V2 had interactions with PperS-RNases, while the hypervariable region of *PperSFB2* HVa-HVb only interacted with PperS2-RNase showed by Y2H and BiFC assay. Bioinformatics analysis of peach genome revealed that there were other F-box genes located at S-locus, and of which three F-box genes were specifically expressed in pollen, named as *PperSLFL1, PperSLFL2*, and *PperSLFL3*, respectively. In phylogenetic analysis *PperSLFLs* clustered with Maloideae *SFBB* genes, and *PperSFB* genes were clustered into the other group with other *SFB* genes of *Prunus*. Protein interaction analysis revealed that the three PperSLFLs interacted with PperSSK1 and PperS-RNases with no allelic specificity. *In vitro* ubiquitination assay showed that PperSLFLs could tag ubiquitin molecules onto PperS-RNases. The above results suggest that three PperSLFLs are the appropriate candidates for the “general inhibitor,” which would inactivate the S-RNases in pollen tubes, involved in the self-incompatibility of peach.

## Introduction

Self-incompatibility (SI) allows the pistil to reject genetically related pollen and promotes out-crossing in flowering plants, which maintains plant genetic diversity (de Nettancourt, 2001). Many plants in Solanaceae, Rosaceae, and Plantaginaceae exhibit S-RNase-based gametophytic self-incompatibility (GSI). GSI is controlled by at least two genes in S-locus: one is pistil-part, a highly polymorphic *S* gene encoding extracellular ribonuclease called S-RNase (Huang et al., 1994), and the other is pollen-part specific *S* gene, which is tightly linked to the *S-RNase* (Entani et al., 2003). The pollen *S* genes of the S-RNase-based GSI are F-box genes called *SLF* (S-locus F-box) in Solanaceae and Plantaginaceae and *SFB* (*S* haplotype-specific F-box) in *Prunus* (Lai et al., 2002; Ushijima et al., 2003; Yamane et al., 2003; Sijacic et al., 2004; Sassa et al., 2010; Tao and Iezzoni, 2010; Meng et al., 2011). S-RNase is secreted into intercellular space of style, and taken up into cytoplasm of compatible and incompatible pollen tubes elongating in style tissues with no choice, and pollen tube stop growing in incompatible crosses due to cytotoxic effects of self S-RNase (Luu et al., 2000; Goldraij et al., 2006; McClure et al., 2011; Boivin et al., 2014). Several studies have proved that *S*-locus F-box proteins act as a subunit of the SCF complex, an E3 ubiquitin ligase, to discriminate between self and non-self S-RNase, and mediate the ubiquitination of non-self S-RNases for degradation by the 26S proteasome (Hua and Kao, 2006, 2008; Lechner et al., 2006; Franklin-Tong, 2008). However, how F-box proteins discriminate between self and non-self S-RNases in pollen tubes of Rosaceae is still largely unknown.

In subfamily Maloideae (e.g., apple and pear) of Rosaceae, polyploidization breaks SI in pollen but does not affect the pistil (de Nettancourt, 2001). The pistil of “Fertility” (2x) could accept pollen from autotetraploid (4x), but “Fertility” (2x) pollen was rejected by the pistil of autotetraploid (4x) (Crane and Lewis, 1942). Genetic analysis reveals that the breakdown of SI can be explained by “competition” between different *S* alleles in pollen. But in *Prunus* (subfamily Prunoideae), tetraploidy is not always associated with self-compitibility. The tetraploid species sour cherry (*Prunus cerasus*) includes both SI and SC plants (Lansari and Iezzoni, 1990). Hauck proposed that the breakdown of SI sour cherry is caused by the accumulation of non-functional *S* haplotypes, rather than competitive interaction in heteroallelic pollen according to the genetic analysis of SI sour cherry (Hauck et al., 2006). In Japanese pear, *S*^4*sm*^ pollen lacking *SFBB1-S*^4^ are rejected by compatible *S*^1^ pistils but accepted by S^3^ and S^5^ pistils (Okada et al., 2004, 2008). On the other hand, loss-of-function of SFBB1-S^5^ had no effect on SI phenotype, and genetic analysis reveals that *S*^5^ pollen is normally accepted by *S*^1^*, S*^3^, and *S*^4^ pistils (Kakui et al., 2011). The fruit set analysis shows that *S*^5^ pollen is normally compatible with *S*^2^ and *S*^9^ pistils and rejected by *S*^5^ pistils (Kajiura et al., 1967, 1969, 1974). On the contrary, in *Prunus*, a truncated SFB protein or lacked the *SFB* gene can confer pollen-part self-compatibility (Ushijima et al., 2004; Sonneveld et al., 2005; Hauck et al., 2006; Tsukamoto et al., 2006; Tao et al., 2007). That is to say, pollen specifity is determined by one *S*-locus F-box gene known as *SFB* (S-haplotype specific F-box gene) in *Prunus* (Entani et al., 2003; Ushijima et al., 2003; Sonneveld et al., 2005), while multiply F-box genes located at the *S*-locus determining Pyreae (Malus, Pyrus, and Sorbus) pollen specificity called *SFBBs* (*S*-locus F-box brothers genes) (Sassa et al., 2007; Kubo et al., 2010; Kakui et al., 2011), the different numbers of *S*-pollen genes implied different mechanisms of self-pollen recognition in *Prunus* and Pyreae. So researchers speculate that S-RNase-based GSI seems to consist of two types, according to the different mode of action of pollen S, a “non-self recognition by multiple factors” system and a “self-recognition by a single factor” system (Kakui et al., 2011). The S-RNase-based GSI of *Prunus* represents “self-recognition by a single factor.” In pollen tubes of *Prunus*, a “general inhibitor” inactivates the cytotoxic effect of non-self S-RNases, while a “blocker” molecule protects the self S-RNase specifically to arrest pollen tube growth (Luu et al., 2001; Sonneveld et al., 2005). Although the “general inhibitor” is a hypothetical protein and had been considered to be F-box proteins encoded by *SLFLs* (S locus F-box genes with the low allelic sequence polymorphism) in *Prunus avium* (Matsumoto and Tao, 2016), and in peach, whether the “general inhibitor” is F-box proteins encoded by Pper*SLFL* genes as with in *P. avium* needs further investigation.

Skp1, Cullin1 (CUL1), Rbx1, and F-box proteins together constitute the SCF complex, E3 ubiquitin ligases. The E3 ubiquitin ligase can make substrate proteins polyubiquitination and degrade by the 26S proteasome system. In the SCF complex, the F-box protein determines substrate specifically, Skp1 serves as an adaptor to connect the variable F-box protein and CUL1 protein, CUL1 forms a core catalytic scaffold with Rbx1, and Rbx1 can bind to E2 and catalyzes the transfer of ubiquitin chains from E2 to the substrate protein to make ubiquitination of substrate proteins (Wu et al., 2000; Zheng et al., 2002; Deshaies and Joazeiro, 2009). SLF/SFBB is shown to be a compoment of the SCF complex to detoxified non-self S-RNases. In petunia, SLF is proved to form the SCF complex with Skp1-like and CUL1-p in pollen (Zhao et al., 2010; Entani et al., 2014; Liu et al., 2014), and in Maloideae, SFBB was also shown to form SCF complex which targeted selectively S-RNase and polyubiquitinated it *in vitro* (Yuan et al., 2014).

In our study, we selected 37 representative varieties according to the evolution route of peach, and identified their *S* genotypes. By Y2H and BiFC analyses, we found that PperSFB2 distinguished self S2-RNase from non-self S-RNases by the C-terminal hypervariable region. According to the genome wide analysis, we cloned three F-box genes (*SLFL*) in the *S*-locus, and did some experiments and analysis to determine whether the function of the SLFL proteins is the same as that of *P. avium*. Our results showed that *PperSLFLs* and *PperSFBs* were not clustered into one branch in the phylogenetic tree, while *PperSLFLs* had closer relationship with *SFBB*. PperSLFL proteins had interactions with all the four PperS-RNases with no *S* allelic specificity, and could participate in self-incompatibility of peach as a subunit of SCF complex.

## Materials and methods

### Plant material

Thirty-seven peach varieties (Supplemental Table 1) were selected from the Zhengzhou Fruit Research Institute, Chinese Academy of Agricultural Sciences, Henan Province, China. Peach organs/tissue samples (leaves, styles and pollen) were collected, frozen in liquid nitrogen and stored at −80°C Ultra-low temperature refrigerator for later use.

### DNA and RNA extraction

Peach genomic DNA was isolated from young leaves using the CTAB method (Li et al., 2009), and incubated with RNase I (Invitrogen, CA, USA) at 37°C for 2 h to remove RNA. Total RNA samples were isolated from leaves, styles and pollen using a modified CTAB method (Li et al., 2009) and treated with DNase I (Invitrogen, CA, USA) to remove DNA contamination. RNA was used as template to synthesize first-strand cDNA using the SuperScript reverse transcriptase (Invitrogen, CA, USA) and Oligo-dT primers (According to manufacturer's instructions, Invitrogen, CA, USA).

### PCR for *S* genotype analysis

Peach genomic DNA was used as templates for PCR with the primers listed in Supplemental Table 2. The primers were designed according to the length of the second intron of the *S*-RNases described previously (Tao et al., 1999). The different *S-RNases* and *S* genotypes of peach varieties could be distinguished depending on the size of the amplified fragments.

### Cloning of *PperS-RNases, PperSFBs, PperSLFLs, PperSSK1, PperCUL1*, and *PperRbx1*

Pollen cDNA was used as template to clone *PperSFBs, PperSLFLs, PperSSK1, PperCUL1*, and *PperRbx1* and style cDNA was used as template to clone *PperS-RNases* with the gene specific primers listed in Supplemental Table 2. The PCR products were purified and individually ligated to the pMD19-Tsimple vector (TaKaRa). The constructed vectors were transformed into *E. coli* competent DH5α cells (Transgene biotech, Beijing, China). Each gene selected 3 positive clones for sequencing.

### Tissue-specific expression analysis

cDNA samples synthesized from total RNA from leaves, styles and pollen of the 37 peach varieties included in this study were used as templates to analyze tissue-specific expression of *PperS-RNases, PperSFBs*, and *PperSLFLs*. Gene specific primers were designed and listed in Supplemental Table 2, and the β-actin gene was used as an internal control for constitutive expression with the following thermal cycling condition: a denaturation step at 94°C for 5 min followed by 30 cycles of 95°C for 30 s, 60°C for 30 s, 72°C for 60 s, and then 72°C for 10 mins.

### Construction of the phylogenetic tree of F-Box, CUL1, and SSK1

64 CDSs of *S*-locus F-box genes (Supplemental Table 3) from *Malus domestica, Pyrus pyrifolia, Pyrus bretschneideri, P. avium, Prunus dulcis, Prunus mume, Prunus salicina, Prunus armenica* were used to construct phylogenetic trees with the F-box genes specifically expressed in pollen cloned from 37 peach varieties in this study. The deduced amino acid sequences of Skp1-like proteins and cullin-like proteins from *Arabidopsis thaliana, Antirrhinum hispanicum, Prunus tenella, Petunia integrifolia, Pyrus bretchneideri, M. domestica, P. avium, Prunus persica. P. integrifolia, Vitis vinifera, Nicotiana tabacum, Prunus tomentosa*, and *P. mume* were aligned by CLUSTALW (Supplemental Table 3). Based on the alignment, phylogenetic trees were constructed using MEGA 6.0 program (Tamura et al., 2013) with the neighbor-joining method (Saitou and Nei, 1987) and the bootstrap test replicated 1,000 times.

### Yeast two-hybrid (Y2H) analysis

Yeast transformation and activity of β-galactosidase assays were performed following the manufacturer's instructions (Clontech, CA, USA). The partial CDSs of *PperS-RNases* removed signal peptides and the full-length CDSs of *PperSSK1, PperCUL1*, and *PperPA1* were cloned into pGBKT7 vector (Clontech), whereas the full-length CDSs of *PperSFBs, PperSLFLs, PperSSK1*, and *PperRbx1* were cloned into pGADT7 vector (Clontech).

In order to explore how PperSFB protein differentiates self PperS-RNase from non-self S-RNases, normal *PperSFB2* was divided into four parts: box domain, box-V1, variable regions V1-V2 and hypervariable regions HVa-HVb. The four parts of *PperSFB2* was cloned into pGADT7 vector. Y2H assay was performed to observe the interactions between different portions of PperSFB2 and all PperS-RNases cloned in this study.

For the Y2H assay, AH109 cells containing both AD and BD plasmids were grown on SD/-Leu/-Trp medium for 3 d at 30°C. Ten independent clones for each combination were streaked on SD/-adenine/-His/-Leu/-Trp medium and grown for 3–4 d at 30°C. Then yeast grown on SD/-adenine/-His/-Leu/-Trp medium was stained with X-α-gal (TaKaRa Bio). To quantify the interaction strength, β-galactosidase activity assay was performed using o-nitrophenyl-β-D-galactopyranoside (Sigma Aldrich) as a substrate.

### Bimolecular fluorescence complementation (BiFC) analysis

The pCambia1300 vector was used to construct BiFC fusion vectors, which contained the N- or C-terminal of yellow fluorescence protein (YFP) fragments (YFPN and YFPC), respectively. The full-length CDSs of *PperSLFLs* without stop codon were cloned into pCambia1300-YFPN vectors, whereas the partial CDSs of *PperS*-RNase without stop codon and signal peptide were cloned into pCambia1300-YFPC. All the construct vectors were transformed into *Agrobacterium tumefaciens* GV3101 respectively and co-infiltrated into *Nicotiana Benthamiana* leaves. YFP fluorescence was observed in epidermal cell layers of *N. Benthamiana* leaves after infiltrated 5 days using Olympus BX61 fluorescent microscope (Olympus FluoView FV1000).

The box, box-V1, V1-V2, and HVa-HVb frames without the stop codon were cloned into the pCambia1300-YFPN vectors. The recombinant plasmids containing the *box-YFPN, box-V1-YFPN, V1-V2-YFPN*, or *HVa-HVb-YFPN* fusion gene and *PperS1-RNase-YFPC, PperS2-RNase-YFPC, PperS2m-RNase-YFPC*, or *PperS4-RNase-YFPC* without signal peptides fusion genes and the control plasmid with *YFPN* and *YFPC* were co-transformed into maize (*Zea mays* Linn.Sp.) protoplasts respectively according to Ren et al. (2011). YFP fluorescence was observed using Olympus BX61 fluorescent microscope (Olympus FluoView FV1000). The primers used were listed in Supplemental Table 2.

### Purification of tagged fusion proteins

His-tagged proteins were purified as previously described (Meng et al., 2014). The CDSs of the *PperS-RNases* without signal peptide were cloned into the pEASY-E1 vector (From TransGen Biotech Company) and transformed into the *E. coli* strain BL21 plysS (DE3) (From TransGen Biotech Company). The cells were inoculated into LB medium containing 100 μg/ml ampicillin and placed in a shaker for about 3 h with 37°C and 200 rpm. When the cell suspension OD_600_ reached to about 0.5, isopropyl β-D-1-thiogalactopyranoside (IPTG) was added into medium with the final concentration 0.2 mM to induce protein production. The cell suspension was incubated in the shaker for 10–12 h with 16°C and 180 rpm. His-tagged fusion proteins were purified using Ni-NTA His binding resin (Novagen, USA) as previously described (according to the manufacturer's instructions of Ni-NTA His Bind Resins, Novagen). The full-length coding sequences of pollen-expressed *PperSFB1m, PperSFB2m, PperSFB2, PperSFB4m PperSLFL1, PperSLFL2*, and *PperSLFL3* were cloned into pMAL-c5x vector, which is designed to generate maltose-binding (MBP) fusion proteins. Similarly, the *PperCUL1, PperSSK1*, and *PperRbx1* were cloned into pGEX4T-1 vector, which is designed to produce glutathione S-transferase (GST) fusion proteins. All the GST-fusion proteins and MBP-fusion proteins were purified using glutathione resin and maltose resin as previously described, respectively (Yuan et al., 2014).

### *In vitro* ubiquitination analysis of PperS-RNases

*In vitro* ubiquitination analysis was performed as previously described (Yang et al., 2009; Yuan et al., 2014). Each 50 μl reaction mixture containing 50 mM Tris (pH 7.4), 10 mM MgCl_2_, 2 mM dithiothreitol (DTT), 5 mM HEPES, 2 mM adenosine triphosphate (ATP), 0.05% Triton X-100, 10 mM creatine phosphate, 1 unit of phosphokinase, 10 μg ubiquitin, 50 nM E1 (UBA6, *Petunia hybrida*), 1 mM PMSF, 850 nM E2 (UBH6, *P. hybrida*), and aliquots (2 μg) of the recombinant proteins GST-PpSSK1, GST-CUL1, GST-Rbx1, His-S-RNase and one kind of MBP-SLFL fusion protein were incubated at 30°C for 2 h. For detection of PperS-RNases by immunoblot, rabbit anti-S-RNase immunogloblin Gs (lgGs, made by Beijing ComWin Biotech Company) were used as primary antibodies at a dilution of 1:2,000, and goat conjugated anti-rabbit lgG (Bio-Rad) was used as a secondary antibody at a dilution of 1:10,000. For detection of MBP-PperSLFL proteins and GST fusion proteins by immunoblot, mouse anti-MBP and anti-GST monoclonal antibody (Bio-Rad) were used as primary antibodies at a dilution of 1:3,000, and goat conjugated anti-mouse lgG (Bio-Rad) was used as a secondary antibody at a dilution of 1:10,000. The reaction mixtures without PperS-RNase or PperSLFL proteins were negative controls.

## Results

### Identification of *PperS-RNase* and *PperSFB* alleles in 37 peach varieties

**Figure 1:**
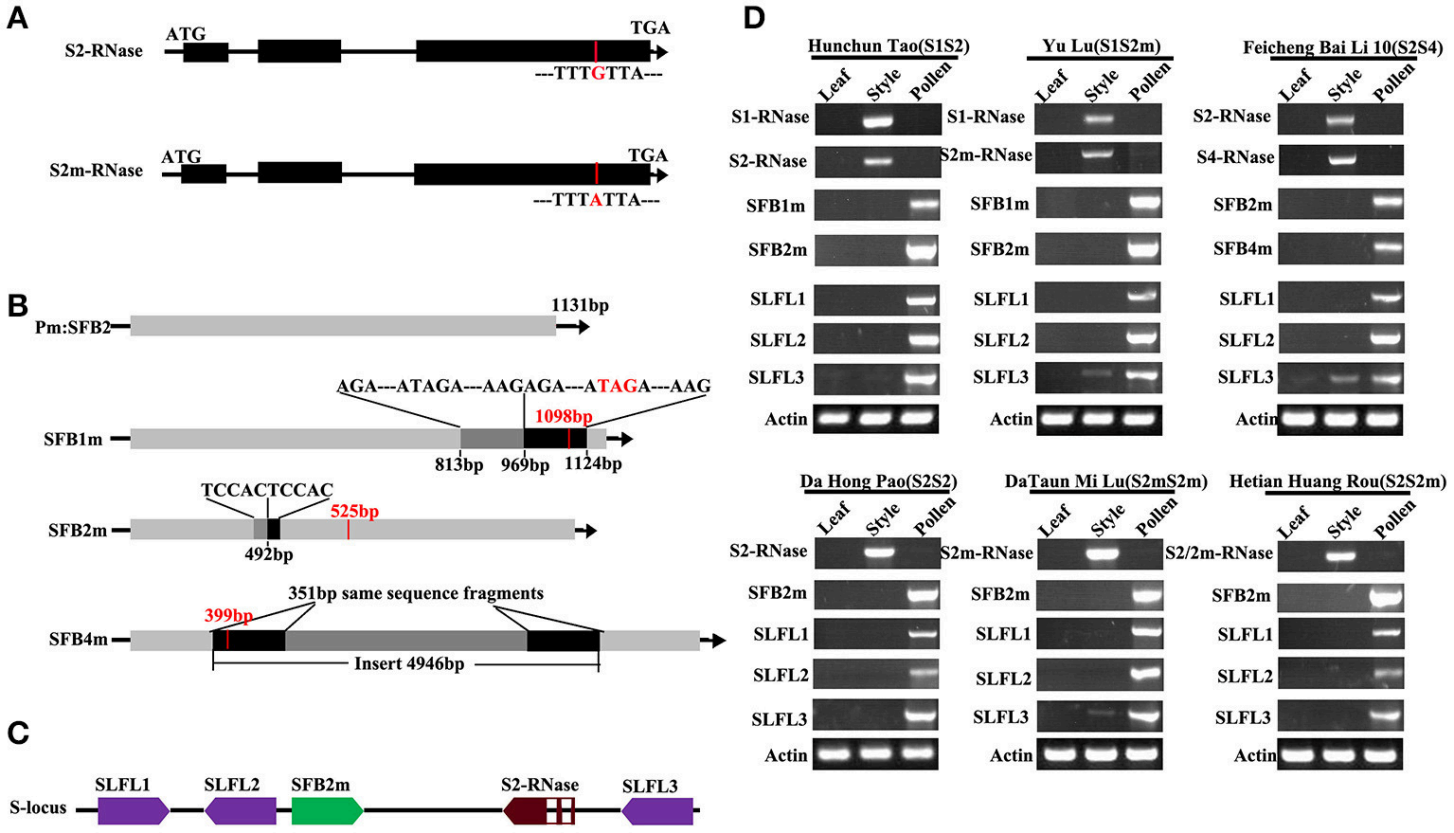
Characterization and expression patterns of peach *S* genes. **(A)** Schematic diagrams of the *S2/2m-RNase*. The red line represents the mutation site of the *S2-RNase*. **(B)** Schematic diagram of peach *PperSFBs*. The black arrows indicate the transcriptional orientations of the genes. The red vertical bars indicate the stop codon, and the red numbers represent the length of the encoding frame. The black boxes in *PperSFB1m* and *PperSFB2m* represent the inserted fragment, and the gray boxes represent the same fragment of the gene as the inserted fragment. The black boxes in *PperSFB4m* represent the same fragments at both ends of the inserted fragment. **(C)** Schematic diagrams of the location of *PperS2-RNase, PperSFB2m*, and *PperSLFLs* at the *S*-locus. The directions of the arrows represent the transcriptional orientations of *PperS2-RNase, PperSFB2m*, and *PperSLFLs*. The middle parts in the red box represent the introns. **(D)** Tissue-specific expression analysis of *PperS1-RNase, PperS2-RNase, PperS2m-RNase* and *PperS4-RNase, PperSFB1m, PperSFB2m, PperSFB4m*, and *PperSLFLs*. Total RNA from different organs was extracted and used as template for cDNA synthesis.

We collected 37 peach varieties which represent local cultivars in 18 provinces/municipalities in China (Supplemental Table 1). These are thought to represent the evolutionary paths of peach from the original regions in central China (Tibet, Yunnan and Guizhou provinces) to the northwest of China (Shanxi province), then to the southwest of China and finally to the coastal and Xinjiang provinces (Cao et al., 2014; Supplemental Figure 1). Only four S-haplotypes *S1, S2, S2m*, and *S4*, were detected from 36 peach varieties except “Guang He Tao” by PCR and sequencing (Supplemental Figure 2). The four *S*-haplotypes have been reported (Tao et al., 2007). The *S* genotype of 18 varieties, including “Da Hong Pao,” were *S2S2* genotype and 9 varieties, including “Hunchun Tao,” were *S1S2* genotype, while 3 varieties were *S2S4* genotype (“Feicheng Bai Li 10,” “Feicheng Bai Li 17,” and “Feicheng Hong Li 6”) (Supplemental Table 1). The *PperS2-RNase* in 6 varieties was observed to contain a nucleotide substitution (G–A), which resulted in the conversion of the sixth conserved cysteine residue to a tyrosine in the *Prunus* C5 domain (Figure 1A). The mutated *PperS2-RNase* was named as *PperS2m-RNase*, which has been reported (Tao et al., 2007). *S2-RNase* and *S2m-RNase* were also identified in the original species “Guang He Tao,” indicating that the mutation of *S2-RNase* had occurred before the formation of peach cultivars. The 4 *PperS-RNases* were specifically expressed in pistil, supporting their roles of pistil determinants (Figure 1D).

The mutated *PperSFBs* cloned from all varieties in this study were the same as previously reported mutations (Tao et al., 2007), and all the *PperSFBm* genes were terminated prematurely. However, we noticed that the sequence of inserted 155 bp fragment in *PperSFB1m* was exactly the same as the sequence of 155 bp fragment upstream of the insertion point. Similarly, the sequence of 5 bp insertion in *PperSFB2m* was also the same as the 5 bp upstream of the insertion point. In addition, the sequences of 351 bp at both ends of the inserted 4,949 bp fragment in *PperSFB4m* were also totally identical (Figure 1B). The repeat sequences of inserted fragment of *PperSFB4m* have been reported (Hanada et al., 2014). In addition, except the mutated *SFB2m*, a canonical *SFB2* gene was cloned from “Guang He Tao,” indicating that peach mutations occurred prior to peach introduction and acclimatization by human and the canonical *SFB2* was eliminated during the selection process. Moreover, the expression of *PperSFBs* was pollen-specific, supporting their role of pollen determinants (Figure 1D).

### Cloning and expression analysis of *PperSLFLs* and members of SCF complex (*PperCUL1, PperSSK1*, and *PperRbx1* genes)

According to the sequences of *S*-locus F-box-likes genes *(SLFLs)* in peach genome (Genome Database for Rosaceae, http://www.rosaceae.org/), the primers were designed to clone the six *SLFL* genes. Finally, only three *SLFL* genes were specifically expressed in pollen (Figure 1D), and we named them as *PperSLFL1, PperSLFL2*, and *PperSLFL3*, respectively. *PperSLFL1* located at about 47 kb downstream of *PperS2-RNase* and the translation direction was opposite to that of *PperS2-RNase*; *PperSLFL2* located at about 26 kb downstream of *PperS2-RNase* and *PperSLFL3* located at about 1.3 kb upstream of *PperS2-RNase*, and the translation direction of *PperSLL2* and *PperSLFL3* was the same as that of *PperS2-RNase*. The three *PperSLFL* genes did not have introns (Figure 1C). The identity of the predicted amino acid sequences of *PperSLFL1, PperSLFL2*, and *PperSLFL3* was 52.45%. All the PperSLFL proteins contained the basic F-box domain and the FBA domain (Supplemental Figure 3). The phylogenetic tree analysis showed the three *PperSLFL* genes clustered with other *Prunus SLFL* genes, which has been reported (Aguiar et al., 2015). The phylogenetic tree had two large lineages. The *Prunus SFB* genes did not cluster with the pollen *S* genes of Pyrus and Malus and clustered into a separate lineage. *Prunus SLFL* genes clustered with Pyrus and Malus *SFBB* genes in another lineage. This suggested that the evolutionary relationship between *Prunus SLFL* genes and *SFBB* genes of Pyrus and Malus was closer than that of between *Prunus SLFL* genes and *Prunus SFB* genes (Supplemental Figure 4A).

The full-length coding sequence (CDS) of the candidate *SSK1* gene (*PperSSK1*) was cloned from “Hunchun Tao” (*S1S2*) pollen and subsequently identified in the other 36 peach varieties with specific primers (Supplemental Table 2). The canonical Skp1 protein comprises 150–200 amino acid residues and contains a Skp1-POZ domain at the N terminus and a Skp1 domain at the C terminus. The deduced amino acid sequence of PperSSK1 comprised 177 residues and contained the Skp1-POZ and the Skp1 domain. In the phylogenetic tree, PperSSK1 clustered into a lineage with PtSSK1 and PavSSK1 (Supplemental Figure 4B). In addition, the other two subunits of the SCF complex, *PperCUL1* and *PperRbx1*, were cloned with pollen cDNA of “Hunchun Tao” (*S1S2*) as template. The deduced PperCUL1 protein containing 744 amino acid residues and phylogenetic analysis showed that it clustered with PavCULB, which have been shown to be a component of the SCF complex (Matsumoto and Tao, 2016) (Supplemental Figure 4C). PperRbx1 protein contained 117 amino acid residues and had an H2 loop figure domain at the C-terminus, which is necessary for ubiquitin ligase activity.

### The interactions between S-RNases and *S*-locus F-box protiens of peach

**Figure 2:**
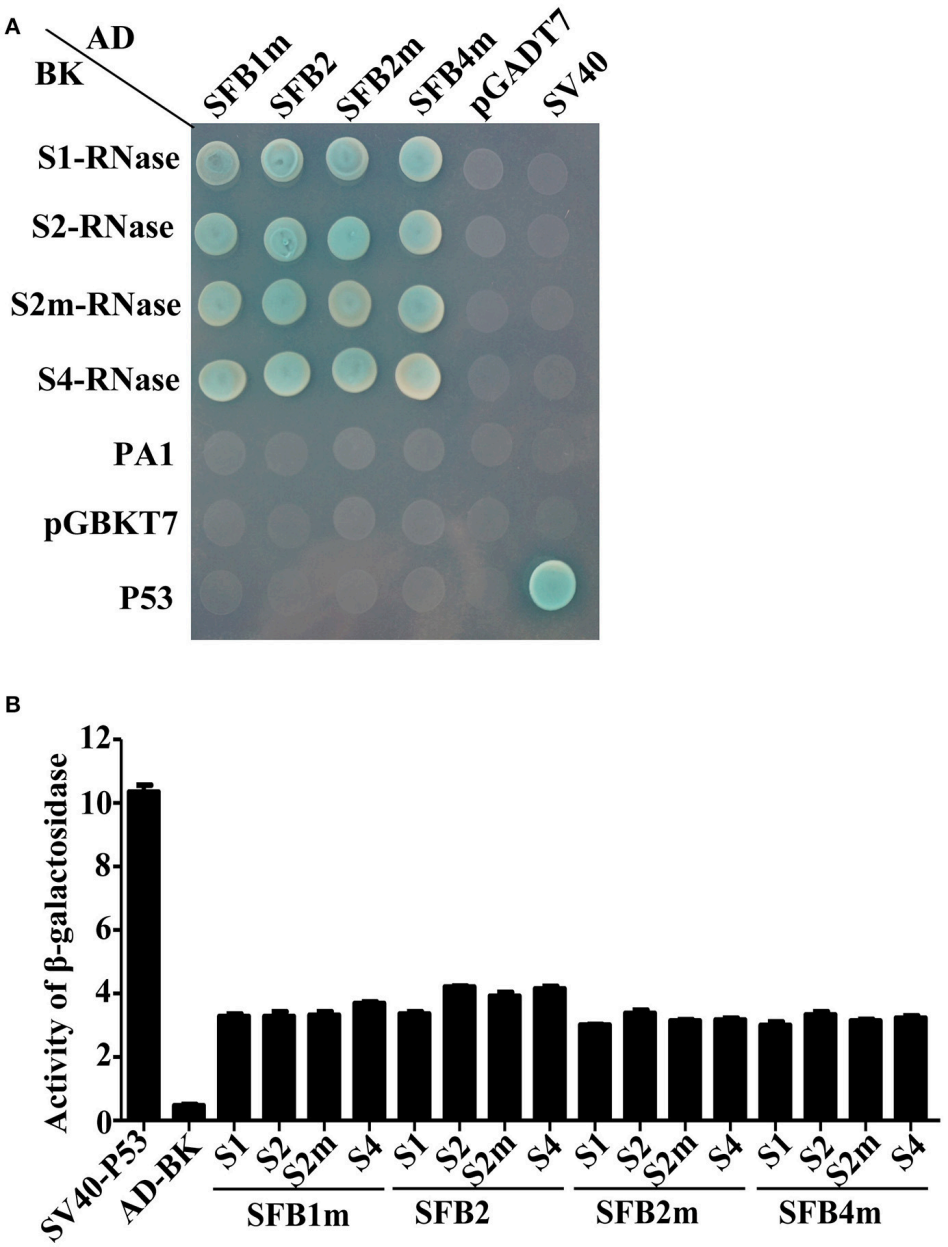
Yeast two-hybrid (Y2H) analysis to investigate interaction between S-RNases and PperSFBs. **(A)** Y2H anlysis for the interactions between PperSFBs and PperS-RNases, PperPA1 (ppa011133m, a T2-RNase in *Prunus persica*). **(B)** Activity of β- galactosidase analysis for the interactions between PperSFBs and PperS-RNases. Empty vector was used as negative control; SV40 and p53 was used as positive. AD, activation domain; BD-DNA, binding domain.

**Figure 3:**
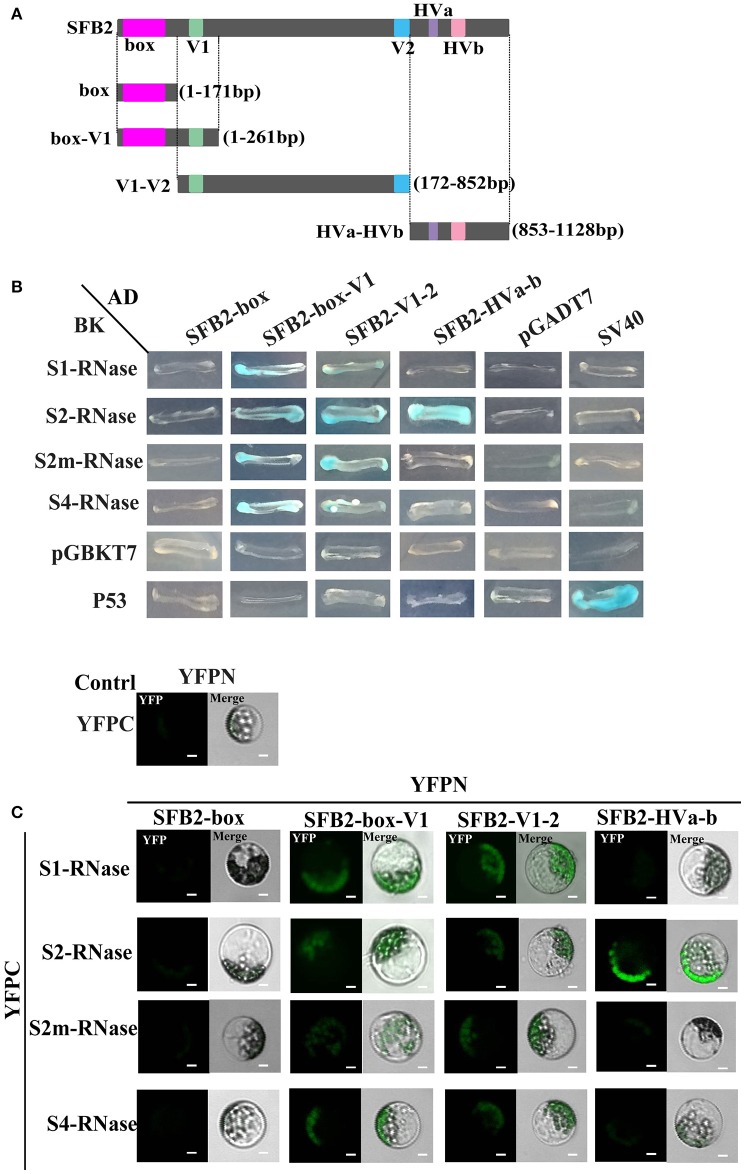
Y2H and BiFC assay for the interactions between PperS-RNases and portions of PperSFB2. **(A)** Schematic diagrams of *PperSFB2* gene segments. Constructs of AD::box, AD::box-V1, AD::V1-V2, and AD::HVa-HVb. **(B)** Y2H analysis for the interaction between PperS-RNases and portions of PperSFB2. Various combinations of AD and BD fusions are tested for their growth on SD/–Leu/-Trp/-His/-Ade media. **(C)** BiFC assay for the interaction between PperS-RNases and portions of PperSFB2. YFPN and YFPC are transiently co-transformated into maize protoplasts. Fluorescence is indicated by the YFP signal. Merged images of YFP as well as bright field images are shown. Scale bars = 10 μm.

Y2H assay was performed to detect the interactions between PperSFBs and PperS-RNases. *PperPA1* (ppa011133m) (Aguiar et al., 2015), also a T2-RNase of *P. persica* with no signal peptide, was cloned into pGBKT7 to detect the interactions with *S*-locus F-box proteins. The results showed that the mutated PperSFBs and normal PperSFB2 interacted with all the PperS-RNases cloned in the study, and these interactions displayed no *S* allelic specificity, while there was no interaction between PperPA1 and PperSFBs (Figure 2A). Furthermore, the activity of β-galactosidase was detected and the results showed that the intensity of interactions between these combinations was not high and the intensity of interaction between normal PperSFB2 and PperS2-RNase was slightly higher than that of other combinations (Figure 2B). Because of the insertion of the fragments, the proteins encoded by *PperSFB1m, PperSFB2m*, and *PperSFB4m* genes were terminated prematurely and the domains at C-terminus was lost in varying degrees. In order to explore the effect of each part of the SFB on S-RNase, we divided the normal *PperSFB2* gene into four parts: box, box-V1, V1-V2, and HVa-HVb (Figure 3A). Y2H and BiFC analysis showed that the box region did not interact with all four PperS-RNases, whereas box-V1 and V1-V2 portions of PperSFB2 physically interacted with the four PperS-RNases, and the interactions displayed no *S* allelic specificity. The yeast coloring time stained by X-α-gal and fluorescence intensity suggested that the interaction intensity of each combination was not high. Interestingly, the HVa-HVb of PperSFB2 only interacted with PperS2-RNase, indicating a potential role in S-RNase-SFB specific recognition (Figures 3B,C).

**Figure 4:**
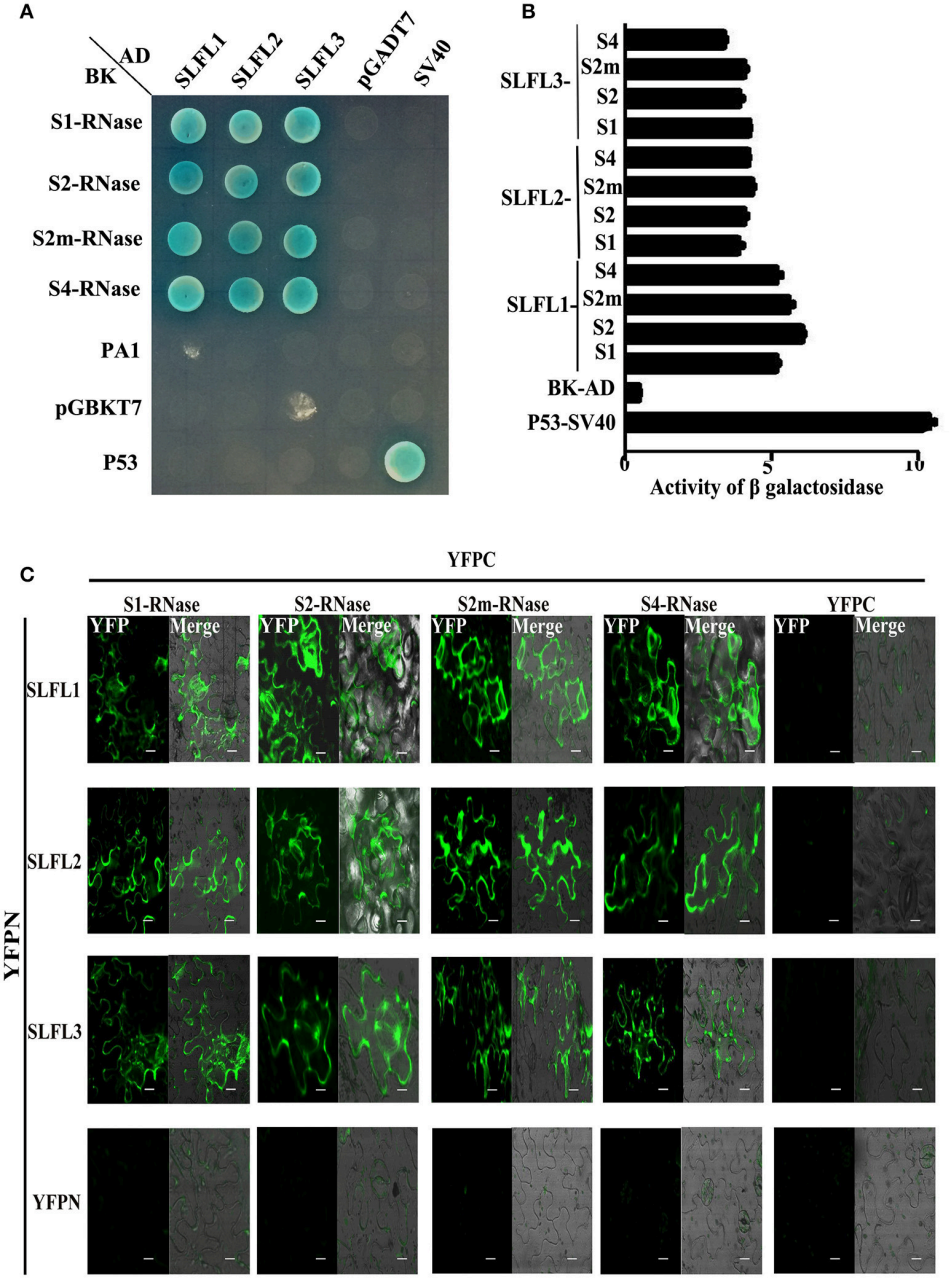
Y2H analysis and BiFC analysis to investigate the interactions between PperSLFLs with PperS-RNases. **(A)** Y2H analysis to investigate the interactions between PperSLFLs and PperS-RNases. **(B)** Activity of β- galactosidase analysis for the interactions between PperSLFLs and PperS-RNases. **(C)** BiFC analysis to investigate the interactions between PperSLFLs with PperS-RNases. Construct pairs of *PperSLFLs-YFPN, PperS-RNases-YFPC*, YFPN, and YFPC are transiently co-infiltrated in *Nicotiana benthamiana* leaves. Fluorescence is indicated by the YFP signal. Merged images of YFP as well as bright field images are shown. *PperSLFLs-YFPN* and *PperS-RNases-YFPC* were co-injected with empty vector respectively as negative control. Scale bars = 10 μm.

The three *PperSLFL1-3* genes specifically expressed in pollen and contained F-box domains, which led to a problem that whether they play roles in self-incompatibility of peach. Firstly, we detected the interactions between PperSLFL1-3 and PperS-RNases. The Y2H assay showed that PperS-RNases interacted with PperSLFL1-3 with no *S* allelic specificity, and PperPA1 did not interact with any PperSLFL proteins (Figure 4A). The activity of β-galactosidase analysis suggested that the intensity of interactions between PperSLFL1 and the four PperS-RNases were slightly higher than other various combinations (Figure 4B). To further confirm the results, BiFC experiment was performed in *N. Benthamiana* leaves, and the results also indicated the interactions between PperSLFL1-3 and the four PperS-RNases with no *S* allelic specificity (Figure 4C).

### Interaction analyses of *S*-locus F-box proteins, PperSSK1, PperCUL1, and PperRbx1

The Y2H analyses were performed to investigate the interactions between PperSSK1 and PperSFBs/PperSLFLs, and the interactions between PperCUL1 and PperSSK1/PperRbx1. The results indicated that PperSSK1 interacted with PperSFB2, PperSFB1m, PperSFB2m, and PperSFB4m (Supplemental Figure 5A), and the activity of β-galactosidase confirmed the intensity of the interaction was high (Supplemental Figure 5B). Both PperSSK1 and PperRbx1 interacted with PperCUL1 (Supplemental Figure 5C), and the activity of β-galactosidase quantitatively demonstrated the interactions between them (Supplemental Figure 5D).

We also examined the interactions between PperSSK1 and three other pollen-expressed F-box proteins, PperSLFL1-3. The Y2H results showed that all the three PperSLFL proteins interacted with PperSSK1 (Supplemental Figure 5A). The activity of β-galactosidase quantitatively demonstrated that the interactions between PperSSK1 and PperSLFL1/PperSLFL2 were stronger than the interaction between PperSSK1 and PperSLFL3, and the interaction between PperSLFL1 and PperSSK1 was the strongest (Supplemental Figure 5B).

### A SCF complex containing PperSLFL1-3 ubiquitinates PperS-RNases *in vitro*

**Figure 5:**
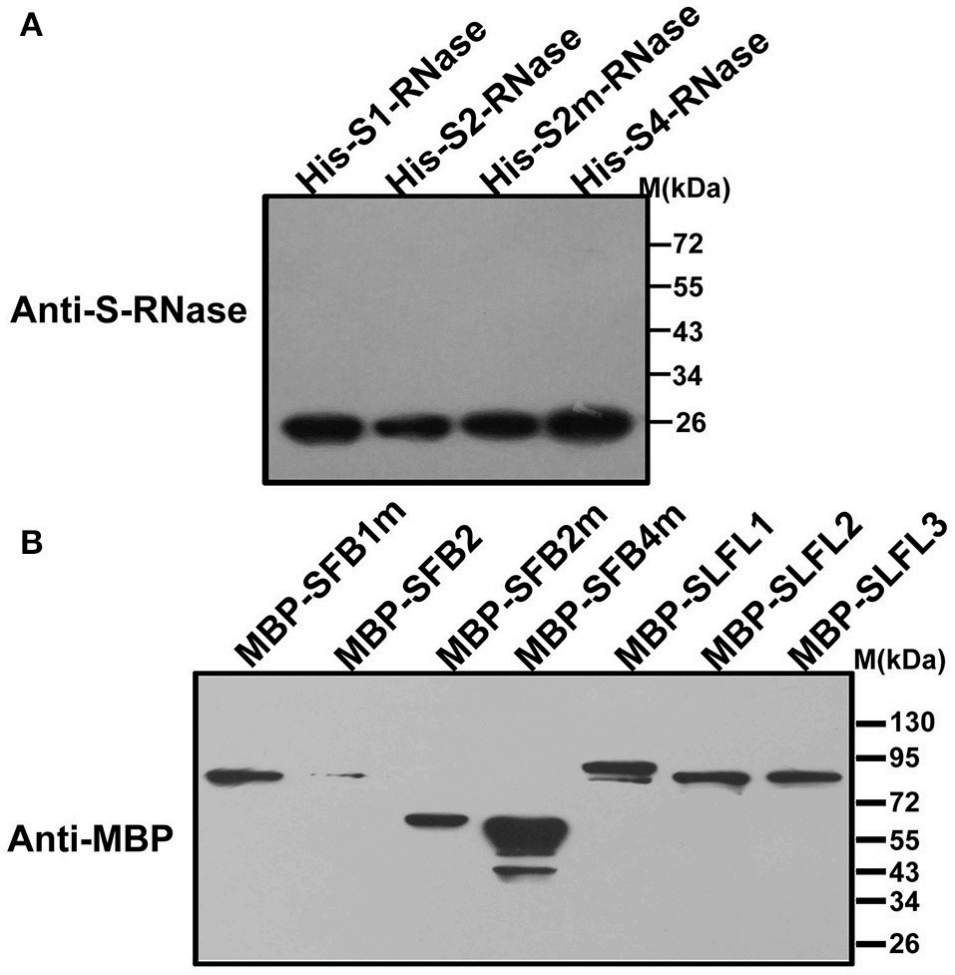
Immunoblot detection of PperS-RNases and F-box proteins **(A)** Immunoblot detection of PperS-RNases. *E. coli* expressed PperS-RNases without signal peptides were detected by polyclonal antibody. The polyclonal antibody against the recombinant PperS2-RNase was raised in rabbit and the antibody detected not only PperS2-RNase but also other allelic PperS-RNases without allelic specificity. **(B)** Immunoblot detection of F-box proteins. *E. coli* expressed F-box proteins were detected by commercial mouse monoclonal antibody. Ten micrograms of total proteins were loaded in each lane.

**Figure 6:**
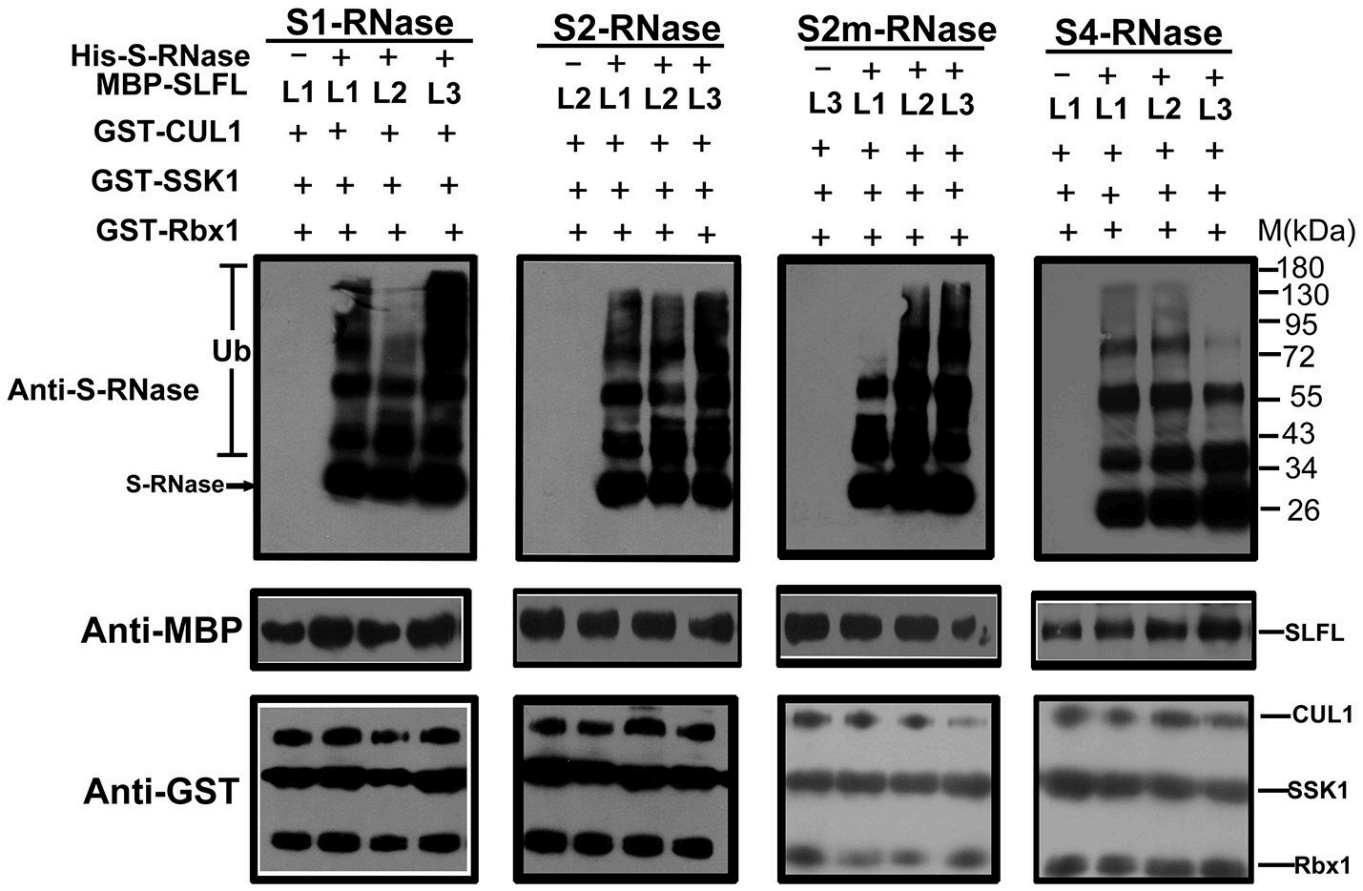
*In vitro* detection of ubiquitinated PperS-RNases. Each lane was loaded with 10 μg protein. The ubiquitination of different PperS-RNases was analyzed with the presence of PperSSK1, PperCUL1, PperSLFL1-3, and Ub. 10 μg of PperSLFL1-3 proteins was added to each lane, respectively. The lanes without His-PperS-RNase were used as negative control. For detection of MBP-PperSLFL proteins and GST fusion proteins by immunoblot, mouse anti-MBP and anti-GST monoclonal antibody (Bio-Rad) were used as primary antibodies.

**Figure 7:**
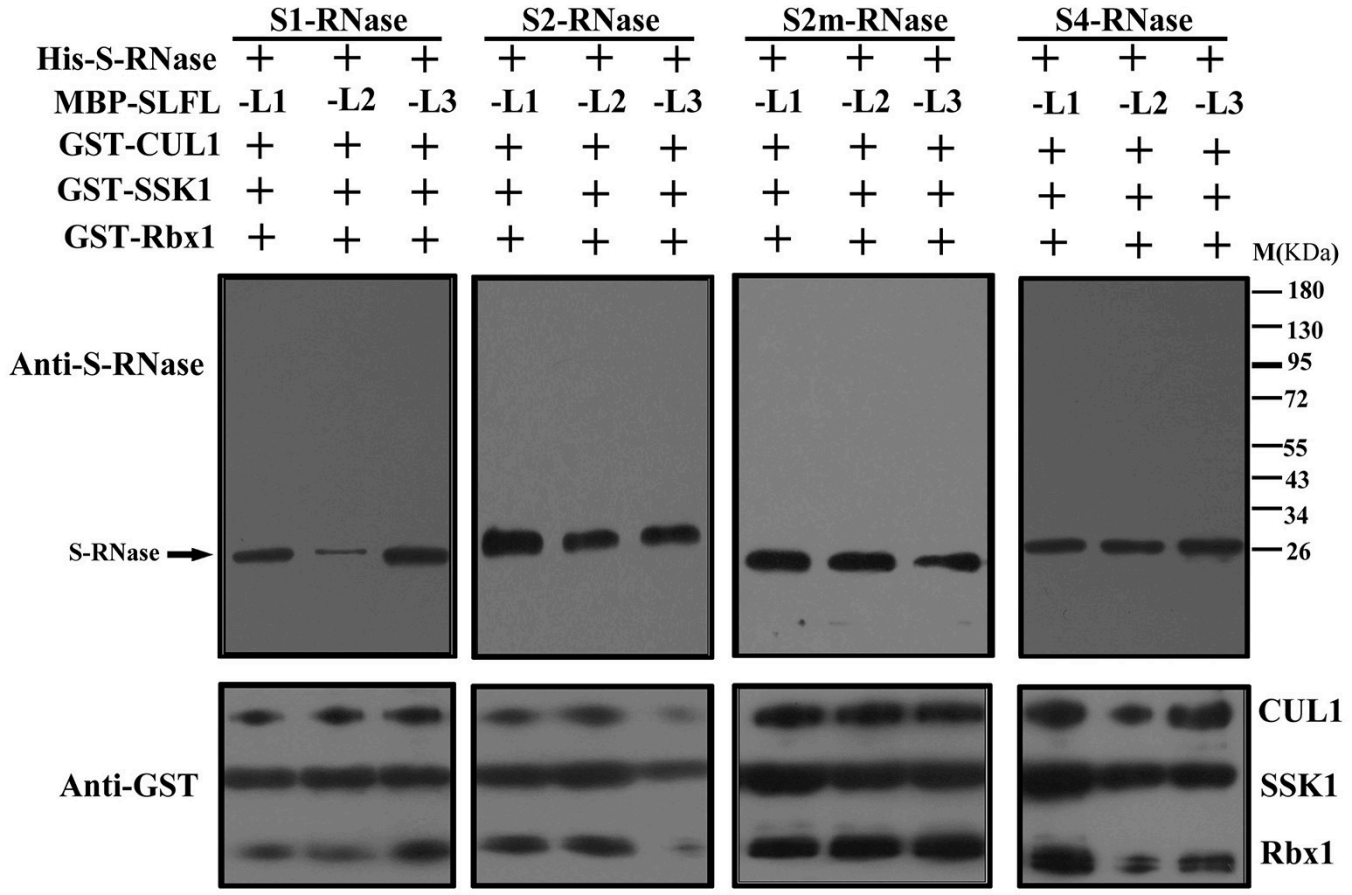
Analysis for ubiquitinated PperS-RNases mediated by SCF complex without PperSLFL proteins. Each lane was loaded with 10 μl reaction solution. PperS-RNase proteins were analyzed by polyclonal antibody against PperS2-RNase raised in rabbit and the antibody detected not only PperS2-RNase but also other allelic PperS-RNases without allelic specificity. GST fusion proteins by immunoblot, mouse anti-GST monoclonal antibody (Bio-Rad) were used as primary antibodies.

In order to test whether PperS-RNases could be ubiquitinated by SCF^SLFL^ *in vitro*, commercial His-UBA6 was used as the ubiquitin-activating (E1) and His-UBH6 was used as ubiquitin-conjugating enzyme (E2). Purified MBP-PperSLFL1-3, GST-PperSSK1, GST-PperRbx1, and GST-PperCUL1 were used as E3. Firstly, the purified His-PperS-RNase proteins, MBP-PperSFB proteins and MBP-PperSLFL proteins were detected, and the results showed that each protein was detected a single band, indicating that these proteins were pure and the antibody was specific (Figures 5A,B). *In vitro* ubiquitination results showed that distinct immunoreactive bands with higher molecular masses (between 34 and 130 KDa) were detected above the predicted His-PperS-RNase (26 KDa) bands. The molecular weight of detected proteins above His-PperS-RNase bands was 8, 16 KDa and so on heavier than that of His-PperS-RNases. The molecular weight of ubiquitin added in the reaction system is 8 KDa, and the extra molecular weight was exactly multiples of ubiquitin molecular weight. No band was detected in the negative control reactions without His-PperS-RNase (Figure 6), and the single 26 KDa protein band was detected in the control reactions without MBP-PperSLFL protein (Figure 7). The results indicated that PperSLFL proteins could ubiquitinate PperS-RNases.

## Discussion

In this study, we selected 37 peach varieties from 18 areas of China, including the ancestral species (Guang He Tao), wild species (Qing Si, Huo Lian Jin Dan, Qing Mao Zi Bai Hua, Bai Nian He, Zhang Bai 5, and Long 1-2-4) and some common local varieties. After identifying the *S* genotype of all the peach varieties in this study (Supplemental Table 1), four previously described *S* haplotypes were identified *S1, S2, S2m* and *S4. S2* was the most frequent *S* haplotype in the selected peach varieties (occurred in 33 varieties), followed by *S1* (in 10 varieties), *S2m* (in 7 varieties) and *S4* (only in 3 varieties). All four *S* haplotypes, *S1 S2, S2m, S4*, found in this study had the same mutant versions as that reported previously (Tao et al., 2007; Hanada et al., 2014). The mutated *S2m-RNase* and *SFB2m* genes, canonical *SFB2* gene and *S2-RNase* existed in “Guang He Tao” indicating that these mutations occurred before the formation of peach cultivars. In the mountains of Tibet, peach might be propagated by seeds generally obtained from genotypes with high productivity. Because of the special natural environment, self-compatibility generally led to more reliable fruit set, which made the probability of survival in natural selection increase, but it was also followed by the decrease of the genetic diversity. We proposed that under selection pressure for SC, pollen part mutants might preferentially be selected compared to pistil part mutants because there were many pollen grains and the pollen genotype in a large extent affected the SI phenotype in GSI system. That may be one reason that all the peach *S* haplotypes in this study are pollen part mutant *S* haplotypes and the *S2*-allele accumulates the most.

After analyzing the sequences of mutant pollen *S* genes, we found that the 155 bp fragment inserted in *PperSFB1m* was duplicated from the 155 bp region upstream of the insertion point, and the 5 bp fragment inserted in *PperSFB2m* was the same with the 5 bp upstream of the insertion point. The sequence of 351 bp at both ends of the inserted 4,949 bp fragment in *PperSFB4m* was also the same that had been reported (Figure 1B) (Hanada et al., 2014). That kind of mutant might be due to an error in homologous recombination, or a retro-transposition, or tandem duplication. As we know, gene duplication is a ubiquitous biological phenomenon, an important driving force for the diversification of genomic and genetic systems, and plays a very important role in the evolution of biological processes. This repetition in peach might be a significant for the study of peach evolution.

S-RNases are degraded by 26S proteasome mediated by SCF^SLF/SFBB^ in other plant species with the S-RNase-based GSI system (Tao and Iezzoni, 2010; Iwano and Takayama, 2012; Yuan et al., 2014). In *Prunus*, two kinds of F-box genes at the *S*-locus: one kind is a single *SFB* gene with high allelic sequence polymorphism, the full name is *S* haplotype-specific F-box and determines pollen specificity, and the other kind is at least three *S* locus F-box genes with low allelic sequence polymorphism, *SLFL* genes, the full names are *S*-locus F-box-like genes (Ushijima et al., 2003). Besides the F-box motif, *SFB* gene contains two variable regions (V1 and V2) and two hypervariable regions (HVa and HVb). V2 and the two hypervariable regions are located in 3′ end of *SFB* gene and are necessary for allele-specific recognition during self-incompatibility reactions (Ushijima et al., 2004). Phylogenetic and evolutionary analysis showed that *PperSLFL1-3* clustered with *SLFL*s of other *Prunus* species in the same evolutionary branch, and the evolution relationship between *SLFL*s and *SFBB*s of apple and pear was closer than that of *SLFL*s and *SFB*s (Supplemental Figure 4A), which was the same as previously reported (Tao et al., 2008). The different numbers of *S* pollen genes implies different mechanisms of self-S-RNase recognition (Romero et al., 2004). Researchers speculated that *Prunus* self-incompatibility mechanism was “self-recognition by a single factor” system (Sonneveld et al., 2005). In the “self-recognition by a single factor” system, the cytotoxic effect of non-self S-RNases is thought to be inactivated by an unidentified “general inhibitor” (GI) (Sonneveld et al., 2005).

Matsumoto et al. (2012), Matsumoto and Tao (2016) showed that PavSFB and PavSLFLs interact with a Skp1-like1 homolog that is proposed to be a component of the SCF complex involved in the polyubiquitination of proteins targeted for degradation (Matsumoto et al., 2012; Matsumoto and Tao, 2016). By Y2H analysis, we have known that PperSLFL1-3 could interact with PperSSK1 and participate in the formation of SCF complex (Supplemental Figures 5A,B). So we speculated that the PperSLFL1-3 would participate in the degradation of PperS-RNase proteins in the process as with SLF/SFBB proteins (Kubo et al., 2010, 2015; Kakui et al., 2011; Williams et al., 2014). There are a lot of F-box proteins in plants. F-box proteins bind to Skp1 through their F-box domain, and the mutants in F-box significantly decrease or abolish binding (Bai et al., 1996). There are also some secondary structures at C-terminal related to interactions between protein-protein, such as leucine zipper (LRR), Kech, WD40, Arm, zinc finger structure and so on, and these domains mediate the specific recognition of the substrate. PperSFBs and PperSLFLSs have F-box motifs, so they have interactions with PperSSK1 (Supplemental Figures 5A,B). Besides the F-box proteins that can form SCF complexes, it has been reported there are several F-box proteins that can form non-SCF complexes (Hermand, 2006). It is possible that PperSFBs form a non- SCF complex with PperSSK1 to play a role in an unknown mechanism to specifically recognize self-S-RNase using their hypervariable regions and protect self-S-RNase cytotoxicity as the “single factor.” PperSFB2 had a role in self/non-self-recognition, the variable regions interacted with self/non-self PperS-RNases, and the hypervariable regions interacted with self-PperS2-RNase (Figures 3B,C). PperSFBs losing hypervariable regions mutations confer SC. It has been reported that a mutation in PavSLFL1 in sweet cherry had no effect on SI response and S-RNases were recognized by PavSLFL2 (Matsumoto et al., 2008; Matsumoto and Tao, 2016). In our study, PperSLFL1-3 interacted with all the PperS-RNases with no *S* allelic specificity (Figure 4). Since it is plausible that the *S*-locus of *Prunus* and the Maloideae share the same origin (Igic and Kohn, 2001; Steinbachs and Holsinger, 2002), we suspect that *SLFLs* are homologs of *SFBB*. During the evolution of SI in *Prunus*, SLFLs may lose their function in *S* haplotype-specific interaction, and recruit SFB for *S* haplotype-specific interaction. *In vitro* ubiquitination analysis, we found that PperSLFL1-3 could make all the PperS-RNase proteins in this study tag the polyubiquitin chain (Figure 6). According to the result, we suspect that the PperSLFL proteins act as GIs to target all PperS-RNases taken up into pollen tubes with no *S* allelic specificity and mediate them to be polyubiquitinated.

In conclution, our results suggested that PperSLFL1-3 were a subunit of SCF complexes, recognized all PperS-RNases taken up into pollen tube and mediated polyubiquitination of PperS-RNases. We speculated that when S-RNases were taken up into the pollen tube, SFB would recognize self S-RNase and protect it by a kind of mechanism, and SLFL proteins could not recognize and target it. Cytotoxic effect of self S-RNase might arrest pollen tube growing. When the SFB mutated, the “protection” on self S-RNase disappeared, SLFL proteins targeted S-RNase and tag polyubiquitin chain on it, S-RNase could be degraded, and the pollen tube continue to grow to complete fertilization. This model is needed to be carefully tested, and further studies are needed to clarify the mechanism of self-incompatibility in *Prunus*.

## Author contributions

TL and DM: designed the study; QC, DM, and ZG: performed the experiment; WL, XD, QY, and YL: contributed reagents/materials; TL and DM: wrote the paper. All authors read and approved the final manuscript.

### Conflict of interest statement

The authors declare that the research was conducted in the absence of any commercial or financial relationships that could be construed as a potential conflict of interest.

## Supplementary material

Supplemental Figure 1 The distribution of 37 peach varieties in China. The arrows in the figure represent the evolutionary direction of peach in China. The red shade represents the origin of peach in China, and the gray shades represent the secondary center of origin of peach in China, and the purple circles represent various peach population.

Supplemental Figure 2 Identification for the S genotypes of 36 peach varieties except Guang He Tao. The PCR were performed with DNA extracted from leaves as template and primers Pru-C2/Pru-C4R for S1 and S2, and S4 specific primers.

Supplemental Figure 3 Alignment of the deduced amino acid sequences of PperSFB2, PperSLFL1, PperSLFL2, and PperSLFL3. The three PperSLFLs and PperSFB2 sequences aligned using DNAMAN. The F-box domain is marked by purple line above, and the FBA domain is marked by black line above.

Supplemental Figure 4 Phylogenetic trees of CDSs of *S* locus F-box and deduced amino acid sequences of Skp1-like proteins and cullin-like proteins. **(A)** Phylogenetic tree of S locus F-box. A neighbor-joining tree was constructed from 64 *S* locus F-box genes from apple (*Malus domestica*; *MdSFBBs*), pear (*Pyrus* × *bretchneideri*; *PbSFBBs. Pyrus pyrifolia*; *PpSFBBs*), sweet cherry (*Prunus avium*; *PavSFBs* and *PavSLFLs*), almond (*Prunus dulcis*; *PdSFBs* and *PdSLFLs*), plum (*Prunus mume*; *PmSFBs* and *PmSLFLs, Prunus salicina*; *PsSFBs*), apricot (*Prunus armeniaca*; *ParSFBs*), sour cherry (*Prunus cerasus*; *PcSFB26*), peach (*Prunus persica*; *PperSFBs* and *PperSLFLs*), and *Prunus speciosa* (*PspSFB1*). **(B)** Phylogenetic tree of Skp1-like proteins. The deduced amino acid sequences of Skp1-like proteins were from *Arabidopsis thaliana* (AtSKPs), *Antirrhinum hispanicum* (AhSSK1), *Prunus tenella* (PtSSK1), *Petunia integrifolia* (PiSKP1,PiSKP3), *Pyrus* × *bretchneideri* (PbSKP1)*, Malus domestica* (MdSSK1-2), *Prunus avium* (PavSSK1) and *Prunus persica* (PperSSK1). **(C)** Phylogenetic tree of cullin-like proteins. The deduced amino acid sequences of cullin-like proteins were from *Arabidopsis thaliana* (AtCUL1-3), *Prunus avium* (PavCUL1A, PavCUL1B), *Petunia integrifolia* (PiCUL1C), *Vitis vinifera* (VvCUL1-1,VvCUL1-2), *Nicotiana tabacum* (NtCUL1-1) *Prunus tomentosa* (PtCUL1), *Prunus mume* (PmCUL), *Pyrus* × *bretchneideri* (PbCUL1, PbCUL1-1), *Malus domestic* (MdCUL1-1,MdCUL1-2), and *Prunus persica* (PperCUL1). NJ trees were generated with 1,000 bootstrap replicates.

Supplemental Figure 5 Yeast two-hybrid analysis and activity of β-galactosidase analysis for the interactions between F-box proteins and PperSSK1, PperCUL1 and PperSSK1, PperRbx1, respectively. **(A)** Yeast two-hybrid analysis for the interactions between F-box proteins and PperSSK1. **(B)** The activity of β-galactosidase analysis for the interactions between F-box proteins and PperSSK1. Each of the combinations was selected 10 yeast plaques and then divided into 3 portions. Each portion was cultured and the activity of β-galactosidase was measured separately. **(C)** Yeast two-hybrid analysis for the interactions between PperCUL1 and PperSSK1, PperRbx1. **(D)** The activity of β-galactosidase analysis for the interactions between PperCUL1 and PperSSK1, PperRbx1. Each of the combinations was selected 10 yeast plaques and then divided into 3 portions. Each portion was cultured and the activity of β-galactosidase was measured separately.

Supplemental Table 1 Thirty-seven wild and local genotypes of Chinese peach.

Supplemental Table 2 Primers for various genes and the constructs.

Supplemental Table 3 The sequence IDs of genes in phylogenetic trees.
