# Supplementary figures and images for: SLFL Genes Participate in the Ubiquitination and Degradation Reaction of S-RNase in Self-compatible Peach
Source: Front Plant Sci. 2018 Feb 22;9:227. doi: 10.3389/fpls.2018.00227 (PMC5826962; doi:10.3389/fpls.2018.00227)

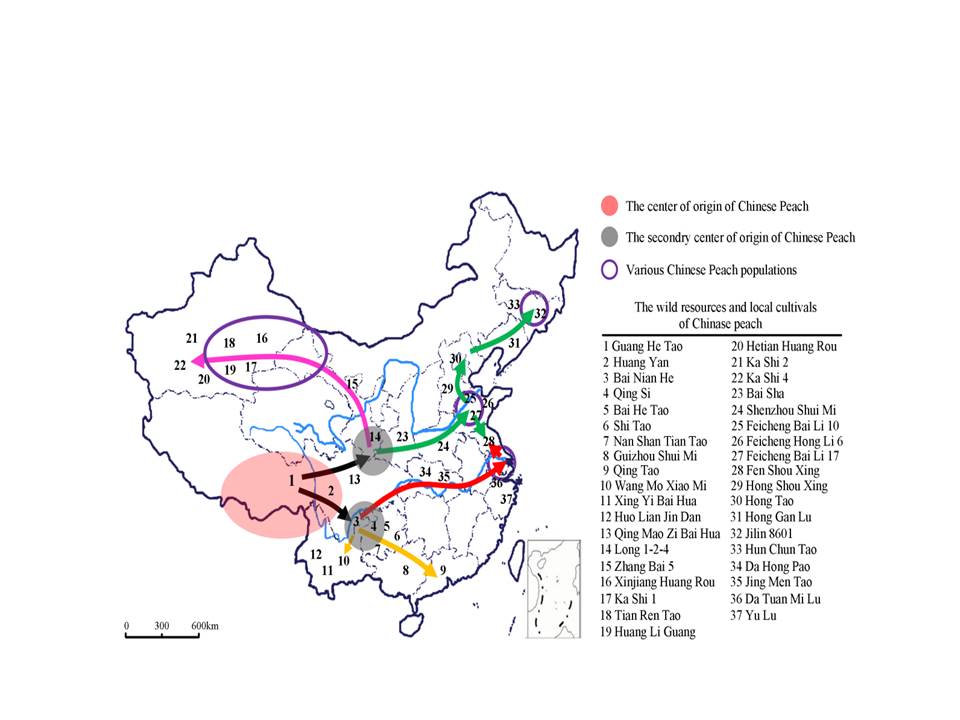

Supplement: Supplemental Figure 1 — The distribution of 37 peach varieties in China. The arrows in the figure represent the evolutionary direction of peach in China. The red shade represents the origin of peach in China, and the gray shades represent the secondary center of origin of peach in China, and the purple circles represent various peach population. [file Image1.JPEG]

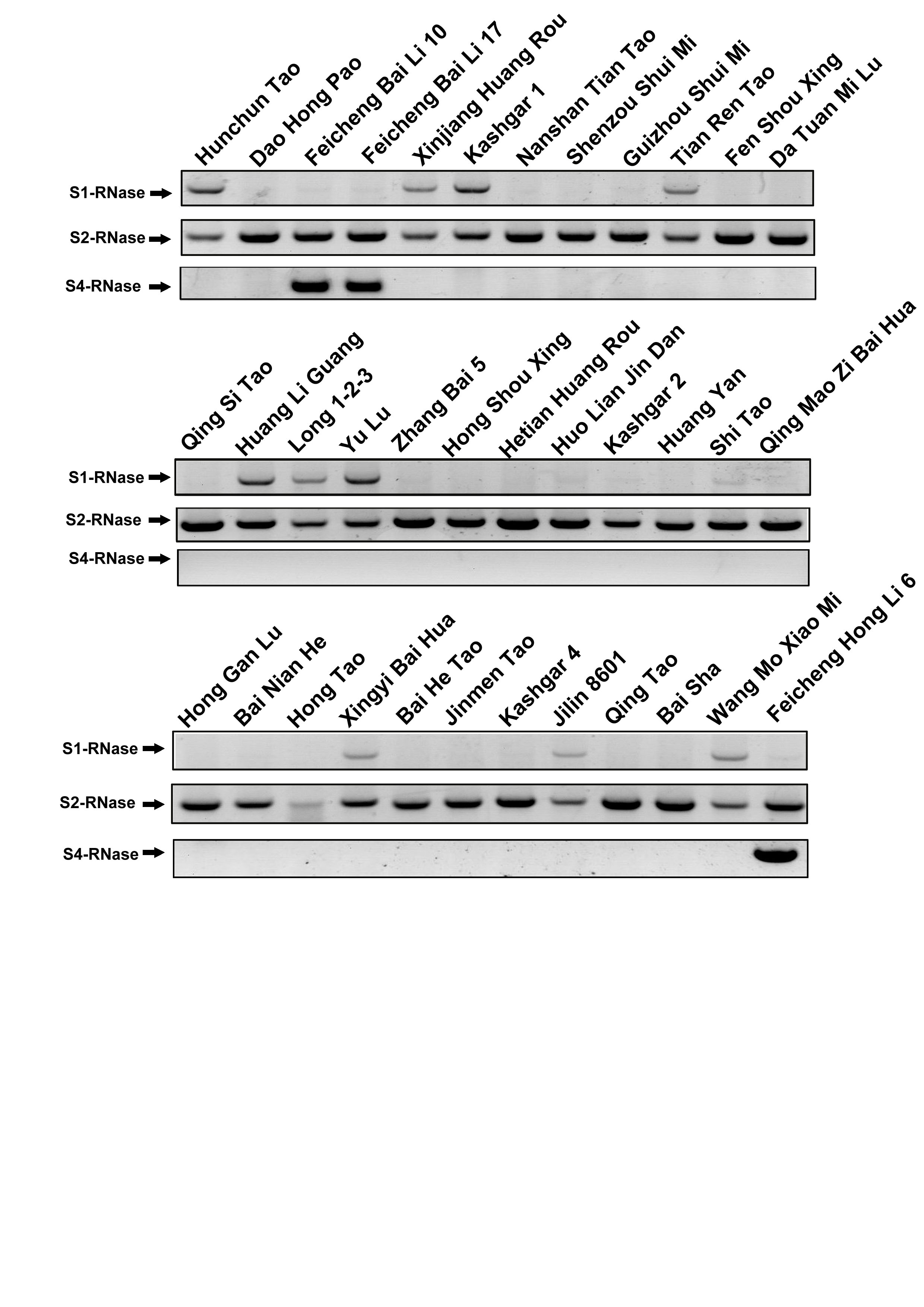

Supplement: Supplemental Figure 2 — Identification for the S genotypes of 36 peach varieties except Guang He Tao. The PCR were performed with DNA extracted from leaves as template and primers Pru-C2/Pru-C4R for S1 and S2, and S4 specific primers. [file Image2.JPEG]

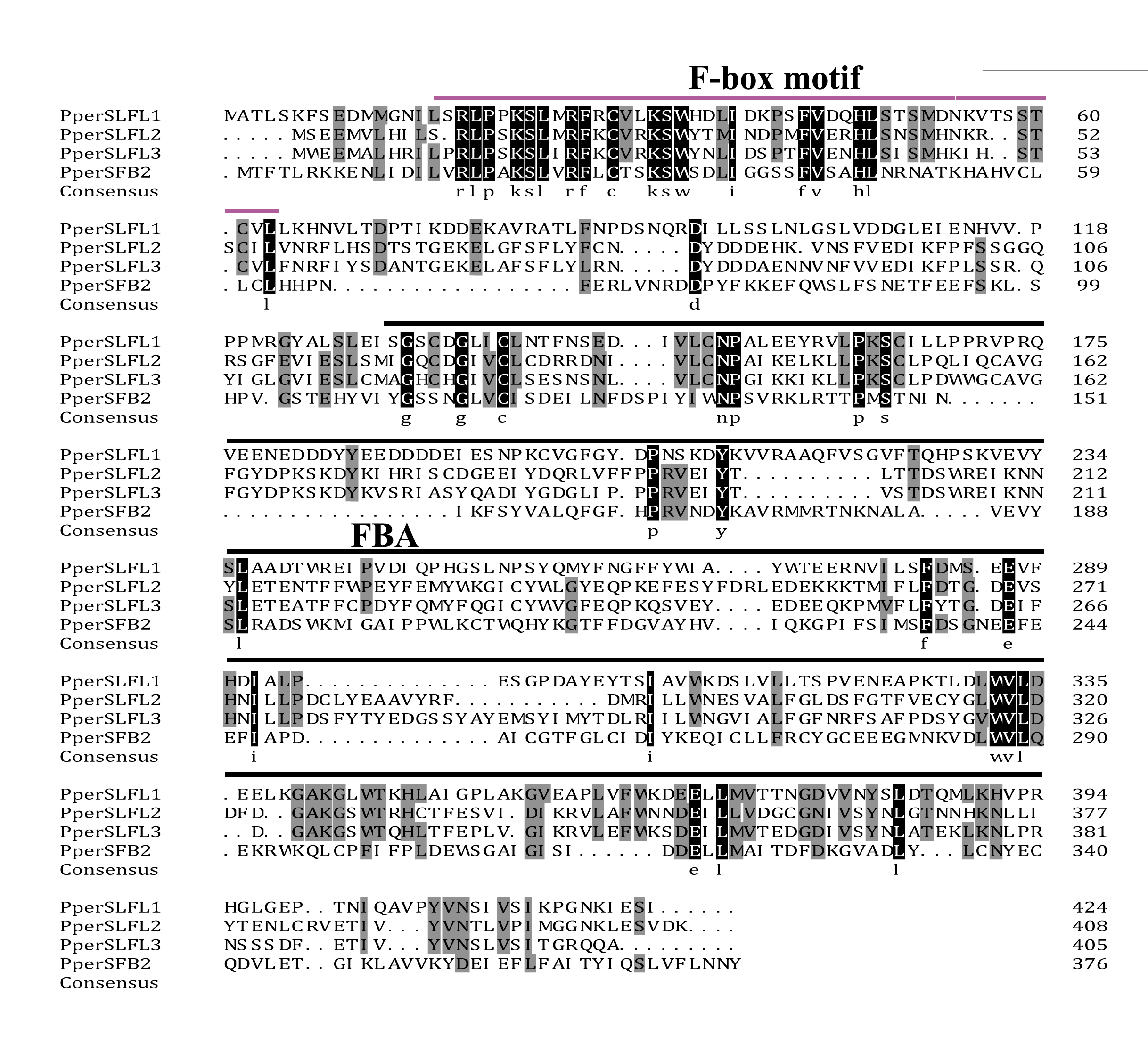

Supplement: Supplemental Figure 3 — Alignment of the deduced amino acid sequences of PperSFB2, PperSLFL1, PperSLFL2, and PperSLFL3. The three PperSLFLs and PperSFB2 sequences aligned using DNAMAN. The F-box domain is marked by purple line above, and the FBA domain is marked by black line above. [file Image3.JPEG]

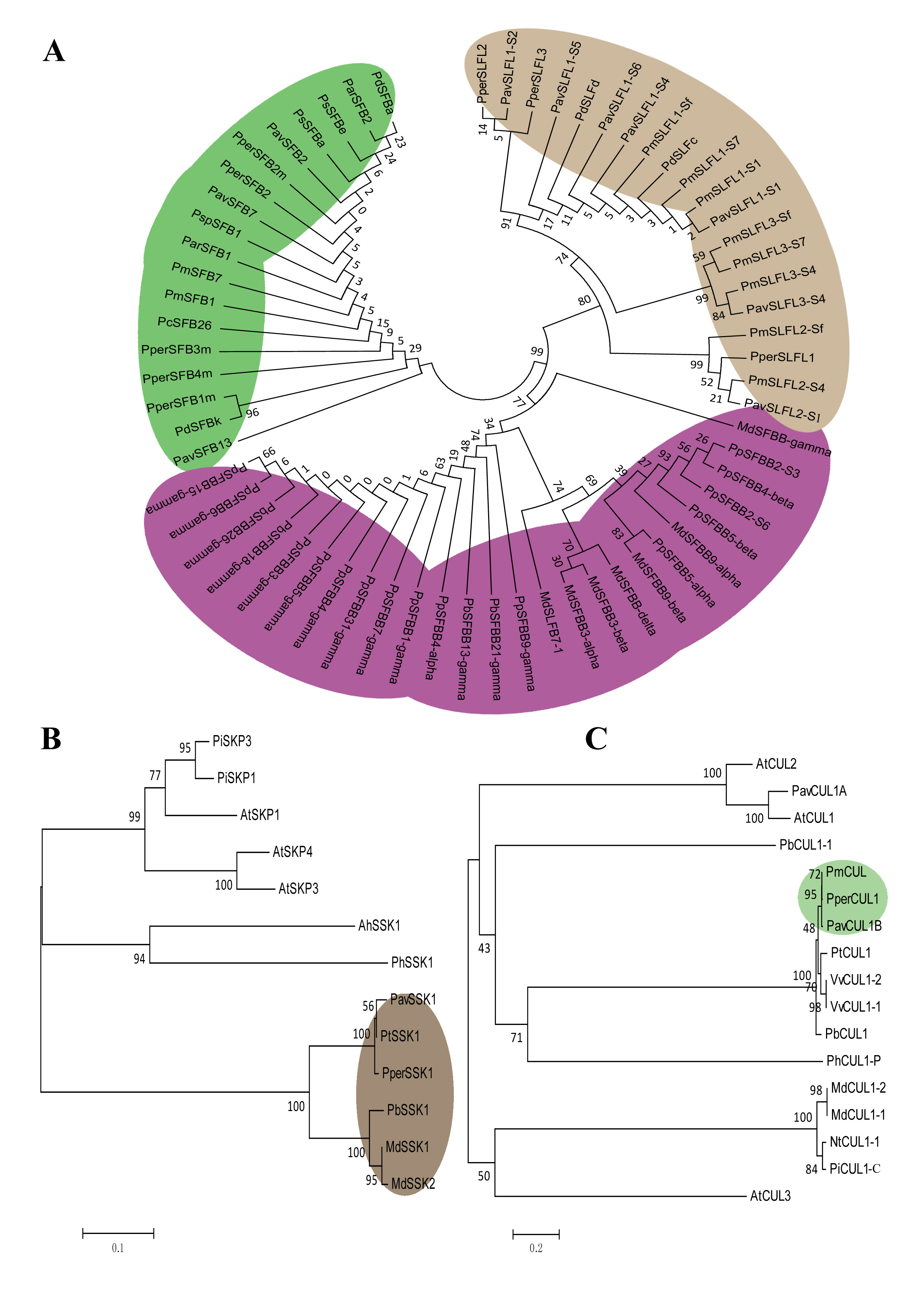

Supplement: Supplemental Figure 4 — Phylogenetic trees of CDSs of S locus F-box and deduced amino acid sequences of Skp1-like proteins and cullin-like proteins. (A) Phylogenetic tree of S locus F-box. A neighbor-joining tree was constructed from 64 S locus F-box genes from apple (Malus domestica; MdSFBBs), pear (Pyrus × bretchneideri; PbSFBBs. Pyrus pyrifolia; PpSFBBs), sweet cherry (Prunus avium; PavSFBs and PavSLFLs), almond (Prunus dulcis; PdSFBs and PdSLFLs), plum (Prunus mume; PmSFBs and PmSLFLs, Prunus salicina; PsSFBs), apricot (Prunus armeniaca; ParSFBs), sour cherry (Prunus cerasus; PcSFB26), peach (Prunus persica; PperSFBs and PperSLFLs), and Prunus speciosa (PspSFB1). (B) Phylogenetic tree of Skp1-like proteins. The deduced amino acid sequences of Skp1-like proteins were from Arabidopsis thaliana (AtSKPs), Antirrhinum hispanicum (AhSSK1), Prunus tenella (PtSSK1), Petunia integrifolia (PiSKP1,PiSKP3), Pyrus × bretchneideri (PbSKP1), Malus domestica (MdSSK1-2), Prunus avium (PavSSK1) and Prunus persica (PperSSK1). (C) Phylogenetic tree of cullin-like proteins. The deduced amino acid sequences of cullin-like proteins were from Arabidopsis thaliana (AtCUL1-3), Prunus avium (PavCUL1A, PavCUL1B), Petunia integrifolia (PiCUL1C), Vitis vinifera (VvCUL1-1,VvCUL1-2), Nicotiana tabacum (NtCUL1-1) Prunus tomentosa (PtCUL1), Prunus mume (PmCUL), Pyrus × bretchneideri (PbCUL1, PbCUL1-1), Malus domestic (MdCUL1-1,MdCUL1-2), and Prunus persica (PperCUL1). NJ trees were generated with 1,000 bootstrap replicates. [file Image4.JPEG]

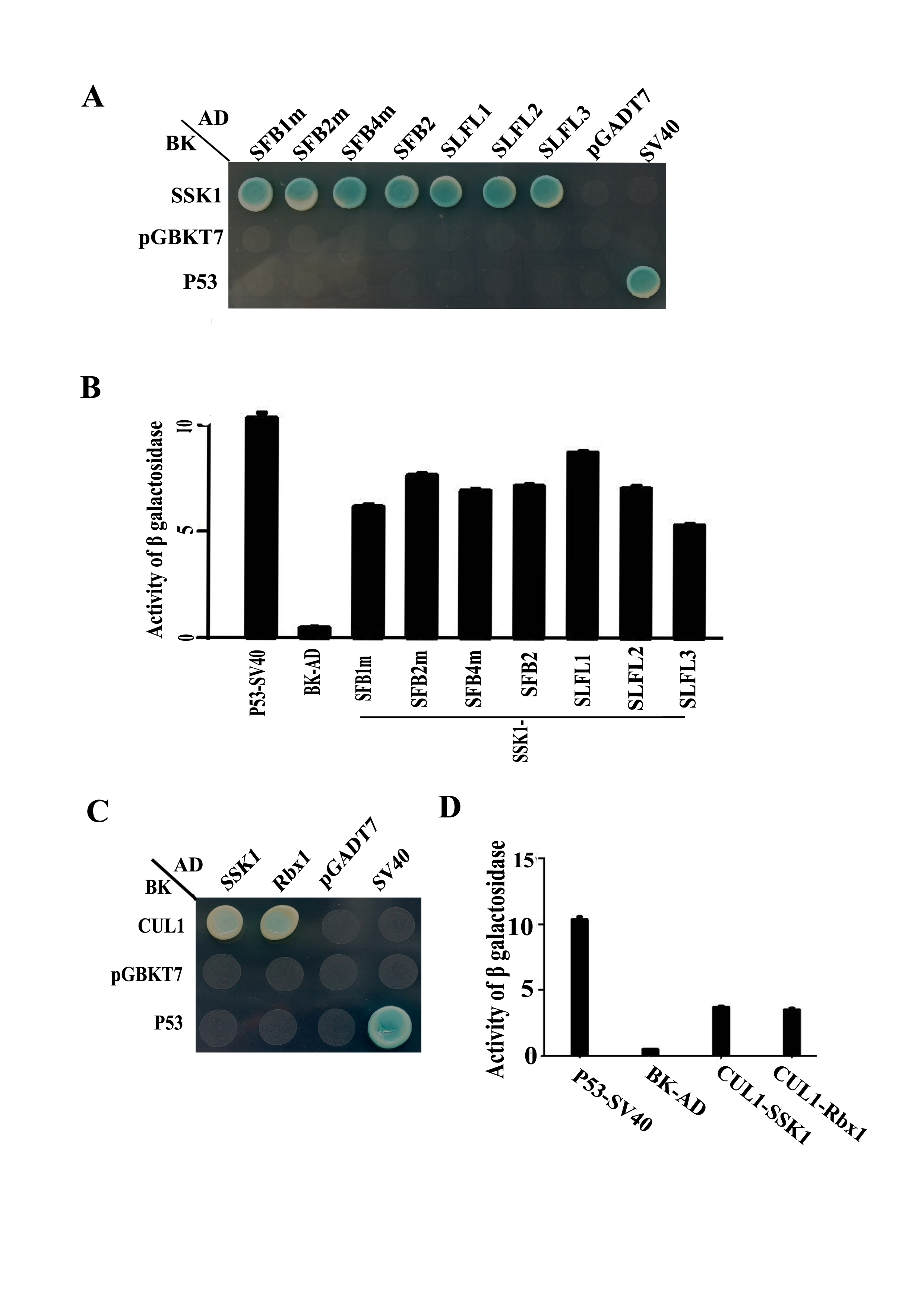

Supplement: Supplemental Figure 5 — Yeast two-hybrid analysis and activity of β-galactosidase analysis for the interactions between F-box proteins and PperSSK1, PperCUL1 and PperSSK1, PperRbx1, respectively. (A) Yeast two-hybrid analysis for the interactions between F-box proteins and PperSSK1. (B) The activity of β-galactosidase analysis for the interactions between F-box proteins and PperSSK1. Each of the combinations was selected 10 yeast plaques and then divided into 3 portions. Each portion was cultured and the activity of β-galactosidase was measured separately. (C) Yeast two-hybrid analysis for the interactions between PperCUL1 and PperSSK1, PperRbx1. (D) The activity of β-galactosidase analysis for the interactions between PperCUL1 and PperSSK1, PperRbx1. Each of the combinations was selected 10 yeast plaques and then divided into 3 portions. Each portion was cultured and the activity of β-galactosidase was measured separately. [file Image5.JPEG]
