# Supplementary material for: SLFL Genes Participate in the Ubiquitination and Degradation Reaction of S-RNase in Self-compatible Peach
Source: Front Plant Sci. 2018 Feb 22;9:227. doi: 10.3389/fpls.2018.00227 (PMC5826962; doi:10.3389/fpls.2018.00227)
Supplement: Supplemental Table 1 — Thirty-seven wild and local genotypes of Chinese peach. [file Table1.DOCX]

| **Supplemental Table 1. Thirty seven wild and local genotypes of peach** | | | | | |
| --- | --- | --- | --- | --- | --- |
| **NO.** | **Cultivar** | **Latin Name** | **Origin (province)** | ***S*-genotype** | **Type of *SFB*** |
|  | **/Accession** |  |  |  |  |
| 1 | Guang He Tao | *Prunus mira* Koehne | Tibet | *S2S2m* | *SFB2/SFB2m* |
| 2 | Huang Yan | *Prunus persica* L. | Yunnan | *S2S2* | *SFB2m/SFB2m* |
| 3 | Bai Nian He | *Prunus persica* L. | Yunnan | *S2S2* | *SFB2m/SFB2m* |
| 4 | Qing Si | *Prunus persica* L. | Yunnan | *S2S2* | *SFB2m/SFB2m* |
| 5 | Bai He Tao | *Prunus persica* L. | Yunnan-Gui Plateau | *S2S2* | *SFB2m/SFB2m* |
| 6 | Shi Tao | *Prunus persica* L. | Guangxi | *S2S2* | *SFB2m/SFB2m* |
| 7 | Nan Shan Tian Tao | *Prunus persica* L. | Guangzhou | *S2S2* | *SFB2m/SFB2m* |
| 8 | Guizhou Shui Mi | *Prunus persica* L. | Guizhou | *S2S2* | *SFB2m/SFB2m* |
| 9 | Qing Tao | *Prunus persica* L. | Guizhou | *S2S2* | *SFB2m/SFB2m* |
| 10 | Wang Mo Xiao Mi | *Prunus persica* L. | Guizhou | *S1S2* | *SFB1m/SFB2m* |
| 11 | Xingyi Bai Hua | *Prunus persica* L. | Guizhou | *S1S2* | *SFB1m/SFB2m* |
| 12 | Huo Lian Jin Dan | *Prunus persica* L. | Sichuan | *S2S2* | *SFB2m/SFB2m* |
| 13 | Qing Mao Zi Bai Hua | *Prunus persica* L. | Sichuan | *S2mS2m* | *SFB2m/SFB2m* |
| 14 | Long 1-2-4 | *Prunus kansuensis* Rehd | Gansu | *S1S2* | *SFB1m/SFB2m* |
| 15 | Zhang Bai 5 | *Prunus kansuensis* Rehd | Gansu | *S2S2* | *SFB2m/SFB2m* |
| 16 | Xinjiang Huang Rou | *Prunus ferganensis* Kost.et Riab | Xinjiang | *S1S2* | *SFB1m/SFB2m* |
| 17 | Kashi 1 | *Prunus ferganensis* Kost.et Riab | Xinjiang | *S1S2* | *SFB1m/SFB2m* |
| 18 | Tian Ren Tao | *Prunus ferganensis* Kost.et Riab | Xinjiang | *S1S2* | *SFB1m/SFB2m* |
| 19 | Huang Li Guang | *Prunus ferganensis* Kost.et Riab | Xinjiang | *S1S2* | *SFB1m/SFB2m* |
| 20 | Hetian Huang Rou | *Prunus ferganensis* Kost.et Riab | Xinjiang | *S2S2m* | *SFB2m/SFB2m* |
| 21 | Kashi 2 | *Prunus ferganensis* Kost.et Riab | Xinjiang | *S2S2* | *SFB2m/SFB2m* |
| 22 | Kashi 4 | *Prunus ferganensis* Kost.et Riab | Xinjiang | *S2S2* | *SFB2m/SFB2m* |
| 23 | Bai Sha | *Prunus persica* L. | Shanxi | *S2S2* | *SFB2m/SFB2m* |
| 24 | Feicheng Bai Li 17 | *Prunus persica* L. | Shandong | *S2S4* | *SFB2m/SFB4m* |
| 25 | Feicheng Bai Li 10 | *Prunus persica* L. | Shandong | *S2S4* | *SFB2m/SFB4m* |
| 26 | Feicheng Hong Li 6 | *Prunus persica* L. | Shandong | *S2S4* | *SFB2m/SFB4m* |
| 27 | Shenzhou Shui Mi | *Prunus persica* L. | Henan | *S2S2* | *SFB2m/SFB2m* |
| 28 | Fen Shou Xing | *Prunus persica L.* | Jiangsu | *S2S2* | *SFB2m/SFB2m* |
| 29 | Hong Shou Xing | *Prunus persica* L. | North China | *S2S2m* | *SFB2m/SFB2m* |
| 30 | Hong Tao | *Prunus persica* L. | Beijing | *S2mS2m* | *SFB2m/SFB2m* |
| 31 | Hong Gan Lu | *Prunus persica* L. | Liaoning | *S2S2* | *SFB2m/SFB2m* |
| 32 | Jilin 8601 | *Prunus persica* L. | Jilin | *S1S2* | *SFB1m/SFB2m* |
| 33 | Hun Chun Tao | *Prunus persica* L. | Jilin | *S1S2* | *SFB1m/SFB2m* |
| 34 | Da Hong Pao | *Prunus persica* L. | Hubei | *S2S2* | *SFB2m/SFB2m* |
| 35 | Jingmen Tao | *Prunus persica* L. | Hubei | *S2S2* | *SFB2m/SFB2m* |
| 36 | Datuan Mi Lu | *Prunus persica* L. | Shanghai | *S2mS2m* | *SFB2m/SFB2m* |
| 37 | Yu Lu | *Prunus persica* L. | Zhejiang | *S1S2m* | *SFB1m/SFB2m* |

The origin, pollen fertility, scientific name, *S*-genotype and types of *SFB* of 37 peach genotypes are shown.
