# Supplementary material for: SLFL Genes Participate in the Ubiquitination and Degradation Reaction of S-RNase in Self-compatible Peach
Source: Front Plant Sci. 2018 Feb 22;9:227. doi: 10.3389/fpls.2018.00227 (PMC5826962; doi:10.3389/fpls.2018.00227)
Supplement: Supplemental Table 2 — Primers for various genes and the constructs. [file Table2.DOCX]

**Supplemental Table 2 Primers for various genes and the constructs**

| Primer name | Forward primer(5'-3') | Reverse primer(5'-3') |
| --- | --- | --- |
| Pru-C2/C4R | CTATGGCCAAGTAATTATTCAAACC | GGATGTGGTACGATTGAAGCG |
| S1 | ATGGGGATGC TGAAATCGTC | TTATTGAAACCA GATGGCTTGT TG |
| S2 | TTCTGTTGTATTTCTTGTTGCTTTA | TTTTTATTATCATACTACATTACTA |
| S4 | ATGGCGATGTTGAAACCGTCACT | TTGAAAGGATATGGTTGTTGATTT |
| SFB1m | TATTGCTTCCCATTGCCAAT | AAACTTGCTTGGATTCGTAA |
| SFB2 | TTCTTTTCAGGATGA | TTAATAATTATTGAGGAAAACCAAA |
| SFB2m | TTCTTTTCAGGATGA | CATCCTTACAGCCTT |
| SFB4m | ATTTATTGCCAACCTCAAAG | ACCATTTAACTGACG |
| SLFL1 | CATCTGTGATGTGAGATATATTGTAT | GTAAAATAACATGAGAAAAGGAATAA |
| SLFL2 | AGAGTATAATCCGATCCGATTAAGTTT | TGCTGAATTACTTATCTACACTCTCAA |
| SLFL3 | CTCGTCGTTCAATCATTAGGGTTTAG | TTGGACAATAGGAAAAAGAGAGAGAA |
| SLFL4 | ATGCCAGAAGAAATGGTGGTG | TCAATTAACTGAAATGAGGCTACTT |
| SLFL5 | CATTGAAGCAGAAATGACAGAGC | GCTGCAAACCGGAACATACTC |
| SLFL6 | ATGACAGAGGAAATGGAGGTGC | TTAAAGACACTTGTGATCAAGGGAG |
| SSK1 | ATGTCGGCCGAGGAGGAGAAG | TCAGTCCTCATCAACTCCTTCAAAA |
| CUL1 | ATGACGATGAACGAGCGTAAGAC | TCATGCCAAGTACCTAAACAAATTG |
| Rbx1 | ATGGCGACATTGGATTCAGATG | CTAATGACCATACTTCTGAAACTCC |
| PA1 | TATGAGATATTAGATCATCAGCATG | ATTATTTATAATAGATGTCCGTTCC |
| Actin | GTGACAATGGAACTGGAATGG | AGACGGAGGATAGCGTGAGG |
| M13 | CGCCAGGGTTTTCCCAGTCACGAC | ACGGATAATTTCACACGGA |
|  |  |  |
| BD-S1 | CATATGGCTTTCCTTGTTCTTGC | GAATTCTTATTGAAACCAGATGGCT |
| BD-S2 | CATATGGCTTTCCTTGTTCTTG | GAATTCTCATTGAAACTTAATGTCA |
| BD-S4 | CATATGGCTTTCCTTGTTCTAG | GAATTCTTATTGAAAGGATATGGTT |
| BD-PA1 | GAATTCATGCCATTAAGATATG | CTGCAGTTATTTATAATAGATGTCC |
| BD-SSK1 | GAATTCATGTCGGCCGAGGA | CTGCAGTCAGTCCTCATCAACTCCT |
| BD-CUL1 | CCCGGGAATGACGATGAACG | GTCGACTCATGCCAAGTACCTAAAC |
| AD-SFB1m | ATCGATACATGACATTCACACTACG | GAGCTCCTATACTAATCCCGATTGT |
| AD-SFB2m | ATCGATACATGACATTCACACTACG | GAGCTCTTACAGCCTTGTAGTCATT |
| AD-SFB2 | ATCGATACATGACATTCACACTACG | GAGCTCTTAATAATTATTGAGGAAA |
| AD-SFB4m | GAATTCATGATATTCACACTACG | GGATCCCTCAACATATAGGA |
| AD-SLFL1 | CCCGGGAATGGCAACGTTGAG | GGATCCCTATGCTTTCAATCTTGTT |
| AD-SLFL2 | CCCGGGAATGTCTGAAGAGATG | GGATCCGCTTATCTACACTCTCAAG |
| AD-SLFL3 | CCCGGGCATGTGGGAAGAGAT | GGATCCTAGCTTGTTGCCTCCCTGT |
| AD-SSK1 | GAATTCATGTCGGCCGAGGA | GGATCCTCAGTCCTCATCAACTCCT |
| AD-Rbx1 | GAATTCATGGCGACATTGGATT | GGATCCCTAATGACCATACTTCTGA |
| AD-box | ATCGATACATGACATTCACACTACG | GAGCTCGACATGGGCATGTTTTG |
| AD-box-V1 | ATCGATACATGACATTCACACTACG | GAGCTCAAAAAGAGACCATTGAAAT |
| AD-V1-V2 | ATCGATACTGTCTACTTTGTCTCCA | GAGCTCAACTTTGTTCATGCCCTCC |
| AD-HVa-HVb | ATCGATACGACTTATGGGTTCTGCA | GAGCTCTTAATAATTATTGAGGAAA |
|  |  |  |
| YFPC-S1 | GGATCCGCTTTCCTTGTTCTTG | ACTAGTTTGAAACCAGATGGCTTGT |
| YFPC-S2 | GGATCCGCTTTCCTTGTTCTTG | ACTAGTTTGAAACTTAATGTCAACG |
| YFPC-S4 | GGATCCGCTTTCCTTGTTCTAG | ACTAGTTTGAAAGGATATGGTTTGT |
| YFPN-box | GGATCCATGACATTCACACTACG | ACTAGTGACATGGGCATGTTTTG |
| YFPN-box-V1 | GGATCCATGACATTCACACTACG | ACTAGTAAAAAGAGACCATTGAAAT |
| YFPN-V1-V2 | GGATCCTGTCTACTTTGTCTCCA | ACTAGTAACTTTGTTCATGCCCTCC |
| YFPN-HVa-HVb | GGATCCGACTTATGGGTTCTGCA | ACTAGTATAATTATTGAGGAAA |
| YFPN-SLFL1 | GGATCCATGGCAACGTTGAGC | ACTAGTTATGCTTTCAATCTTGTTG |
| YFPN-SLFL2 | GGATCCATGTCTGAAGAGATG | GTCGACCTTATCTACACTCTCAA |
| YFPN-SLFL3 | GGATCCATGTGGGAAGAGATG | ACTAGTAGCTTGTTGCCTCCCTG |
|  |  |  |
| His-S1 | GCTTTCCTTGTTCTTGCTTTTGCTT | TTATTGAAACCAGATGGCTTGTTGA |
| His-S2 | GCTTTCCTTGTTCTTGTTTTTG | TCATTGAAACTTAATGTCAACG |
| His-S4 | GCTTTCCTTGTTCTAGCTTTTG | TTATTGAAAGGATATGGTTTGTTG |
| MBP-SFB1m | GGATCCATGACATTCACACTACG | CTGCAGCTATACTAATCCCGATTGT |
| MBP-SFB2m | GGATCCATGACATTCACACTACG | CTGCAGTTACAGCCTTGTAGTCATT |
| MBP-SFB2 | GGATCCATGACATTCACACTACG | CTGCAGTTAATAATTATTGAGGAAA |
| MBP-SFB4m | GGATCCATGATATTCACACTACGTA | CTGCAGTCAACATATAGGACTATCG |
| MBP-SLFL1 | GGATCCATGGCAACGTTGAGC | CTGCAGCTATGCTTTCAATCTTGTT |
| MBP-SLFL2 | GGATCCATGTCTGAAGAGATG | CTGCAGGCTTATCTACACTCTCAAG |
| MBP-SLFL3 | GGATCCATGTGGGAAGAGATG | CTGCAGTAGCTTGTTGCCTCCCTGT |
| GST-SSK1 | GGATCCATGTCGGCCGAGGAG | CTCGAGTCAGTCCTCATCAACTCCT |
| GST-CUL1 | CCCGGGTATGACGATGAACGAGCGT | GCGGCCGCTCATGCCAAGTACCTAA |
| GST-Rbx1 | GGATCCATGGCGACATTGGATTCAG | CTCGAGCTAATGACCATACTTCTGA |
