# Supplementary material for: SLFL Genes Participate in the Ubiquitination and Degradation Reaction of S-RNase in Self-compatible Peach
Source: Front Plant Sci. 2018 Feb 22;9:227. doi: 10.3389/fpls.2018.00227 (PMC5826962; doi:10.3389/fpls.2018.00227)
Supplement: Supplemental Table 3 — The sequence IDs of genes in phylogenetic trees. [file Table3.DOCX]

| **Supplemental Table 3 The sequence IDs of genes in phylogenetic trees** | | |
| --- | --- | --- |
| **Gene** | **The gene name** | **Sequence ID** |
| **F-box** | MdSFBB-gamma | HM013921.1 |
|  | MdSFBB-delta | HM013922.1 |
|  | MdSLFB7-1 | GU345816.1 |
|  | MdSFBB3-alpha | AB270795.1 |
|  | MdSFBB3-beta | Ab270796.1 |
|  | MdSFBB9-beta | AB270794.1 |
|  | MdSFBB9-alpha | AB270793.1 |
|  | MdSFBB4-beta | AB270798.1 |
|  | PpSFBB1-gamma | AB297933.1 |
|  | PpSFBB2-S6 | AB621603.1 |
|  | PpSFBB2-S3 | AB621602.1 |
|  | PpSFBB4-alpha(gamma) | AB270799.1 |
|  | PpSFBB7-gamma | AB297937.1 |
|  | PpSFBB5-beta | AB270801.1 |
|  | PpSFBB31-gamma | EU422959.1 |
|  | PpSFBB4-gamma | AB270799.1 |
|  | PpSFBB5-gamma | AB270802.1 |
|  | PpSFBb15-gamma | EU081893.1 |
|  | PpSFBB6-gamma | AB297936.1 |
|  | PpSFBB3-gamma | AB297935.1 |
|  | PpSFBB9-gamma | AB297939.1 |
|  | PpSFBB5-alpha | Ab270800.1 |
|  | PbSFBB18-gamma | EU081894.1 |
|  | PbSFBB26-gamma | EU418248.1 |
|  | PbSFBB13-gamma | EU081892.1 |
|  | PbSFBB21-gamma | EU422961.1 |
|  | PavSFB2 | AB111519.1 |
|  | PavSFB7 | EU035976.1 |
|  | PavSFB13 | DQ385844.1 |
|  | ParSFB1 | AY587563.1 |
|  | ParSFB2 | AY587562.1 |
|  | PdSFBa | AB092966.1 |
|  | PdSFBk | AB252408.1 |
|  | PmSFB1 | AB101440.1 |
|  | PmSFB7 | AB101441.1 |
|  | PsSFBa | AB252410.1 |
|  | PsSFBe | AB280794.1 |
|  | PcSFB26 | EU035977.1 |
|  | PspSFB1 | HM347508.1 |
|  | PperSFB1m | AB252414.1 |
|  | PperSFB2(m) | AB252416.1 |
|  | PperSFB3m | AB537564.1 |
|  | PperSFB4m | AB537566.1 |
|  | PavSLFL3-S4 | AB280955.1 |
|  | PmSLFL3-S7 | AB092627.1 |
|  | PmSLFL3-Sf | AB280958.1 |
|  | PmSLFL3-S4 | AB280955.1 |
|  | PmSLFL1-S1 | AB092623.1 |
|  | PavSLFL1-S2 | AB360340.1 |
|  | PavSLFL1-S6 | AB360342.1 |
|  | PavSLFL1-S1 | AB360339.1 |
|  | PmSLFL1-S7 | AB092624.1 |
|  | PmSLFL1-Sf | AB280956.1 |
|  | PavSLFL1-S4 | AB280953.1 |
|  | PavSLFL1-S5 | AB360341.1 |
|  | PavSLFL2-S4 | AB280954.1 |
|  | PmSLFL2-S1 | AB092625.1 |
|  | PmSLFL2-Sf | AB280957.1 |
|  | PdSLFd | AB101660.1 |
|  | PdSLFc | AB101659.1 |
|  | PperSLFL1 | XM 007207722.2 |
|  | PperSLFL2 | XM 007207906.2 |
|  | PperSLFL3 | XM 007206556.2 |
| **SSK** | AhSSK1 | DQ355480.1 |
|  | AtSKP1 | AY113971.1 |
|  | AtSKP4 | NM 101868.5 |
|  | AtSKP3 | NM 128129.2 |
|  | MdSSK1 | XM 008380451.1 |
|  | MdSSK2 | HE802074.1 |
|  | PavSSK1 | JQ322646.1 |
|  | PtSSK1 | KT984123.1 |
|  | PhSSK1 | FJ490176.1 |
|  | PiSKP3 | DQ250013.1 |
|  | PiSKP1 | DQ250014.1 |
|  | PbSKP1 | HE802072.1 |
|  | PperSSK1 | XM 007212472.2 |
| **CUL** | AtCUL2 | NM 100179.4 |
|  | AtCUL3 | NM 102447.5 |
|  | AtCUL1 | NM 116491.3 |
|  | MdCUL1-2 | XM 008342600.2 |
|  | MdCUL1-1 | XM 008342599.2 |
|  | NtCUL1-1 | XM 016589930.1 |
|  | PhCUL1-P | AB720735.1 |
|  | PiCUL1-C | DQ250016.1 |
|  | PavCUL1A | JQ322649.1 |
|  | PmCUL | XM 008224949.1 |
|  | PavCUL1B | XM 021963021.1 |
|  | PbCUL1 | XM 001302324.1 |
|  | PbCUL1-1 | XM 009353964.2 |
|  | PtCUL1 | XM 002314417.2 |
|  | VvCUL1-2 | XM 010651423.2 |
|  | VvCUL1-1 | XM 002272159.4 |
|  | PperCUL1 | XM 007226930.2 |
